# Polarization-insensitive 3D conformal-skin metasurface cloak

**DOI:** 10.1038/s41377-021-00507-8

**Published:** 2021-04-08

**Authors:** He-Xiu Xu, Guangwei Hu, Yanzhao Wang, Chaohui Wang, Mingzhao Wang, Shaojie Wang, Yongjun Huang, Patrice Genevet, Wei Huang, Cheng-Wei Qiu

**Affiliations:** 1 Air and Missile Defense College, Air Force Engineering University, 710051 Xi’an, China; 2 Institute of Flexible Electronics, Northwestern Polytechnical University, 710072 Xi’an, China; 3 Department of Electrical and Computer Engineering, National University of Singapore, Singapore, 117583 Singapore; 4 School of Information and Communication Engineering, University of Electronic Science and Technology of China, 611731 Chengdu, China; 5 Université Côte d’Azur, CNRS, Centre de Recherche sur l’Hétéro-Epitaxie et ses Applications (CRHEA), 06560 Valbonne, France

**Keywords:** Photonic devices, Microwave photonics

## Abstract

Electromagnetic metasurface cloaks provide an alternative paradigm toward rendering arbitrarily shaped scatterers invisible. Most transformation-optics (TO) cloaks intrinsically need wavelength-scale volume/thickness, such that the incoming waves could have enough long paths to interact with structured meta-atoms in the cloak region and consequently restore the wavefront. Other challenges of TO cloaks include the polarization-dependent operation to avoid singular parameters of composite cloaking materials and limitations of canonical geometries, e.g., circular, elliptical, trapezoidal, and triangular shapes. Here, we report for the first time a conformal-skin metasurface carpet cloak, enabling to work under arbitrary states of polarization (SOP) at Poincaré sphere for the incident light and arbitrary conformal platform of the object to be cloaked. By exploiting the foundry three-dimensional (3D) printing techniques to fabricate judiciously designed meta-atoms on the external surface of a conformal object, the spatial distributions of intensity and polarization of its scattered lights can be reconstructed exactly the same as if the scattering wavefront were deflected from a flat ground at any SOP, concealing targets under polarization-scanning detections. Two conformal-skin carpet cloaks working for partial- and full-azimuth plane operation are respectively fabricated on trapezoid and pyramid platforms via 3D printing. Experimental results are in good agreement with numerical simulations and both demonstrate the polarization-insensitive cloaking within a desirable bandwidth. Our approach paves a deterministic and robust step forward to the realization of interfacial, free-form, and full-polarization cloaking for a realistic arbitrary-shape target in real-world applications.

## Introduction

The research in electromagnetic invisibility has been long pursued and flourished particularly, thanks to the recent developments of metamaterials^1^. One approach is to use transformation optics^2,3^, which essentially consists of designing metamaterials to redirect electromagnetic (EM) waves to flow around a target and thereby renders the target fully invisible. However, this requires to realize complex and even singular constitutive parameters of extreme anisotropy (both electric and magnetic) and inhomogeneity^4–8^, which is very challenging in practice. Another method is the scattering cancelation with plasmonic metamaterials^9–12^ and transmission-line coupled network^13^, requiring sophisticated pairings of each layer to compensate the scattering. Moreover, those devices are usually bulky and not easy to scale up, especially at high frequencies. Recently emerged metasurfaces, a two-dimensional equivalent counterpart of metamaterials, have provided unprecedented capacities to control the amplitude, phase, and polarization of scattering EM waves of a target, which is easy for fabrications and facilitates many fascinating applications^14–26^. The metasurface-enabled cloaking has also been proposed and experimentally achieved recently^27–35^. Therein, by wrapping targets with an elaborately designed metasurface, the reflective phase and amplitude of targets can be engineered to mimic specular reflections at a flat mirror as if the object did not exist. With this strategy, both tunable^36^ and intelligent^37^ cloaks were also developed, further advancing the applicative prospect of metasurfaces for cloaking applications.

Nevertheless, all existing metasurface cloaks are limited to a single or few states of polarization (SOP), being vulnerable if the scatterer introduces complex polarization-conversion scattering or if polarization-scanning signals are used in the detection system. Thereafter, achieving a deterministic conformal-skin full-polarization cloaking is in high demand and yet formidably challenging so far. The difficulties are manifolds. First, a designed artificial meta-atom may exclusively generate dynamic phases for cloaking at a specific SOP, but it would be not likely to perform under another SOP. Second, isotropic meta-atoms (e.g., those with structural rotation symmetry higher than C_3_) have been utilized to demonstrate the full-polarization cloaking, simply because they essentially do not distinguish SOP only under a few linear polarization (LP) states^30,31^. However, the realization of a perfect cloaking interface not only requires the ultimate control on the intensity and wavefront, but also needs to preserve the polarization state of the incident light. For circularly polarized (CP) incident beams, the cloaking effect of the above isotropic strategy would be much diminished at its copolarization component (see Supplementary Section 6). Third, an angle-independent amplitude and phase response is particularly essential for a cloak with arbitrary boundary. The design based on an anisotropic co-LP scheme is far insufficient to preserve the EM response as the symmetry would be broken under oblique large-angle incidences, leading to angle-distinguished responses. As of today, the realization of fully polarized cloaking at Poincaré sphere remains elusive, letting alone that such cloak is even conformal and ultrathin.

**Fig. 1:**
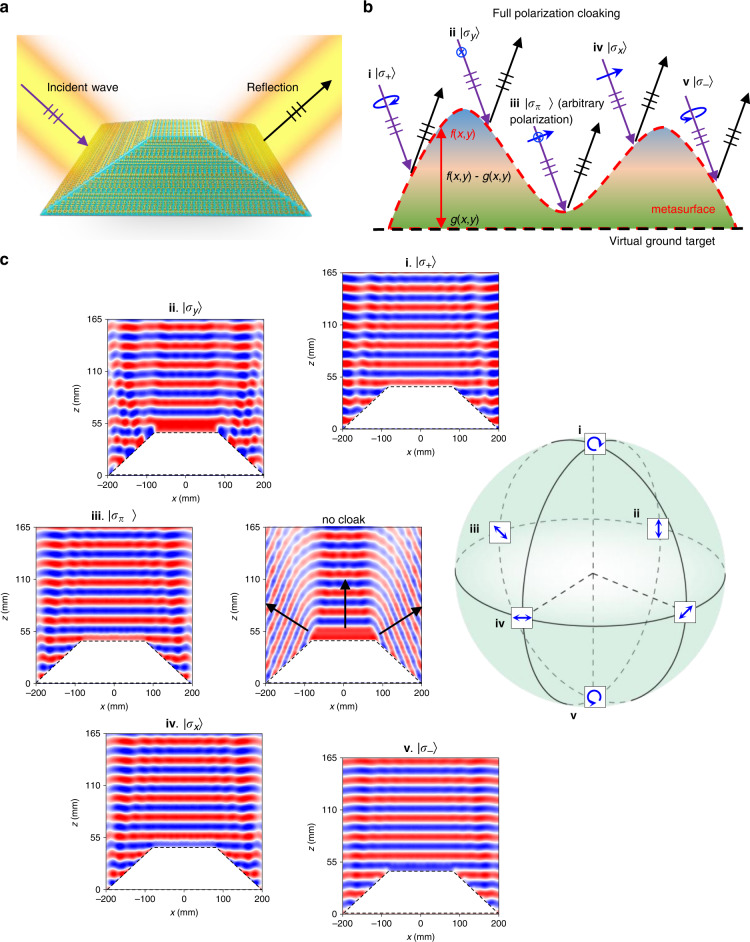
The concept of full-polarization cloaking. **a** Schematic design of a metasurface cloak. **b** The schematic representation of full-polarization metasurface cloak. The arbitrary curved scatter is covered by a metasurface cloak. Under the incidence (purple arrows) of different polarizations, the wavefront (indicated by a triplet short line) can be reconstructed as if the wave experiences a specular reflection (black arrow) at a virtual ground plane. The red dashed line denotes the metasurface cloak at the boundary of scatter denoted by *f*(*x*, *y*); the black dashed line denotes the virtual ground denoted by *g*(*x,y*). **c** The Poincare sphere with different major polarizations denoted from i to v, corresponding to $\left| {\sigma _ + } \right\rangle$, $\left| {\sigma _y} \right\rangle$, $\left| {\sigma _{\pi /4}} \right\rangle$, $\left| {\sigma _x} \right\rangle$, and $\left| {\sigma _ - } \right\rangle$, respectively. The near-field distributions represent the copolarized beam under the normal incidence of a trapezoid metasurface cloak with SOP from i to v and under the incidence of bare bump with SOP of $\left| {\sigma _x} \right\rangle$, accordingly

Here, we report a ubiquitous approach to obtain conformal-skin cloaking for full canonical polarization states described by Poincaré sphere in a half-space reflection scheme (Fig. 1a) with tailored meta-atoms incorporating both dynamic and geometric phases for real-world stealth applications. Importantly, note that the valid cloaking effect for full SOP here does not include the condition of natural light or partially polarized light. Specifically, the wave after impinging onto our metasurface cloak is always precisely deflected to predicted specular reflection directions as if it is grounded by a mirror (Fig. 1b), regardless of incident SOP at any arbitrary point of Poincare spheres (Fig. 1c). The important foundation of full-polarization cloaking is based on synthesizing identical dual-phase patterns in two spin-decoupled channels of an anisotropic conformal-skin metasurface. Our recipe synergizes the cross-LP dynamic and geometric phases based on the sophisticated spin-decoupling theory, which serves as the only option to preserve the output polarization state of our proposed skin metasurface cloak, in both LP and CP modes. It completely addresses the tricky issue of partial polarization insensitivity of existing cloaks under few LP states based on isotropic meta-atoms. It also completely distinguishes it from other polarization-insensitive devices based on anisotropic co-LP geometric phase^38–40^, where an optimization process is necessary to acquire the delicate polarization insensitivity. Moreover, the three-dimensional (3D) cloak is realized by combining a conformal metasurface^41–44^ and a 3D-printing technique^45^, readily extendable to arbitrary complex structures and platforms. This is especially true for the theoretical framework of “conformal boundary optics”, which is very essential to guide conformal design in arbitrary geometries^43,44^. We believe that our approach offers the deterministic polarization-independent cloaking for generally conformal objects with cost-effective fabrications of the skin metasurface and robustness in angle tolerance, which is of great importance for real-world applications.

## Results

### Principle of conformal-skin cloak for full canonical polarization

Without the loss of generality, we start by cloaking a black metallic bump with an arbitrary boundary described by *f*(*x, y*). The purpose of a conformal-skin metasurface cloak is to achieve scattering properties, leading to specular reflection at a ground plane modeled by the contour *g*(*x, y*), as shown in Fig. 1b. By applying ray-tracing techniques, the required compensated phase pattern δ targeted at specific polarization $\left| \sigma \right\rangle$ and wavelength *λ*_0_ can be theoretically calculated as^29^

1 $$\delta _{\left| \sigma \right\rangle}\left({x,y} \right) = \pi - 2k_0\cos \theta \left[ {f\left({x,y} \right) - g\left({x,y} \right)} \right]$$

Here, *k*_0_ = 2π/*λ*_0_ is a free-space wavevector, *θ* is the wave incidence angle with respect to the ground, and $\left| \sigma \right\rangle$ denotes the SOP of the incident beam, which can be found in Poincare spheres, in the form of Jones’ vectors. Note, for simplicity, we also define that $\left\langle {\sigma ^ \bot \left| \sigma \right.} \right\rangle = 0$ where $\langle\left. {\sigma ^ \bot } \right|$ is its cross (orthogonal) polarization. For the sake of clarity, we explicitly define several SOPs here, which are LP with the electric field along *x* ($\left| {\sigma _x} \right\rangle = \left[ {1,0} \right]^{\mathrm{T}}$), along *y* ($\left| {\sigma _y} \right\rangle = \left[ {0,1} \right]^{\mathrm{T}}$), and along the intersection of *x* and *y* axes ($\left| {\sigma _{\pi /4}} \right\rangle = \left[ {1,0} \right]^{\mathrm{T}}$), as well as CP for right-handed CP (RCP, $\left| {\sigma _ + } \right\rangle = \left[ {1/\sqrt 2 ,j/\sqrt 2 } \right]^{\mathrm{T}}$) and left-handed CP (LCP, $\left| {\sigma _ - } \right\rangle = \left[ {1/\sqrt 2 , - j/\sqrt 2 } \right]^{\mathrm{T}}$). In general, the function *g*(*x, y*) can be set to mimic a fictitious target as an illusion cloak for a particular SOP, but for our proof-of-concept demonstrations, we use a constant value to mimic a horizontal ground as carpet cloaking. In the following, we propose the design of a metasurface to provide a wavefront predicted in Eq. (1). Note that the current state of the art in metasurfaces exploits either dynamic or geometric phases to precisely implement the target phase. However, the reflective dynamic-phase discontinuity, described by a Jones matrix $$R = \left(\begin{array}{ll}r_{xx}e^{j\varphi _{xx}} & r_{xy}e^{j\varphi _{xy}}\\ r_{yx}e^{j\varphi _{yx}}&r_{yy}e^{j\varphi _{yy}}\end{array} \right)$$ under the Cartesian coordinate, is commonly associated with the resonant feature of a meta-atom reacting to specific SOP and incident angle. Here, *φ*_*xx*_ (*r*_*xx*_) and *φ*_*yx*_ (*r*_*yx*_) denote the reflective phase (amplitude) of $\left| {\sigma _x} \right\rangle$ and $\left| {\sigma _y} \right\rangle$ components under excitation of $\left| {\sigma _x} \right\rangle$ (the same nomenclature to other parameters). Minimal change of SOP will induce large or complete phase distortions, thus deteriorating the final performance. This makes the full-polarization cloak an extremely difficult problem.

In addition, polarization preservation is another concern of anisotropic co-LP system. Synergizing geometric phase (orientation rotation *α*) and dynamic phase (parametric variation) in an anisotropic co-LP geometry (|$r_{xy}$| = |$r_{yx}$| = 0, $|r_{yy}| = |r_{xx}| = 1$ and $\varphi _{yy} - \varphi _{xx} = 180^\circ$)^46–48^ has been successfully implemented for complete distinct phases at two CP states in terms of $$R^{{\mathrm{CP}}}\left(\alpha \right) = \left(\begin{array}{cc}e^{j\left({\varphi _{xx} - 2\alpha }\right)}& 0\\ 0 & e^{j\left({\varphi _{xx} + 2\alpha } \right)}\end{array} \right) =\left(\begin{array}{cc}r_{\left| {\sigma _+ } \right.\rangle }{e^{j\delta _{\left| {\sigma _+ } \right.\rangle} }} & 0 \\ 0 & r_{\left| {\sigma _- } \right.\rangle }{e^{j\delta_{\left| {\sigma _- } \right.\rangle}}}\end{array} \right)$$_._ However, it is impossible to achieve full-polarization cloaking because the polarization in LP operations cannot be preserved, see Supplementary Section 6. Our proposition, instead, exploits cross-LP dynamic^19,49^ and geometric phases to preserve the output polarization of the conformal-skin cloak in both LP and CP states. Hence, we hereafter adopt a cross-LP scheme (|$r_{xx}$| = |$r_{yy}$| = 0) to decouple phases and functions under $\left| {{\upsigma}_ + } \right\rangle$ and $\left| {{\upsigma}_ - } \right\rangle$ wave (see the generalized theory for both co-LP and cross-LP system in Supplementary Section 1). The Jones’ matrix after a rotation of *α* is formulated as $R^{{\mathrm{LP}}}\left(\alpha \right) = S^{ - 1}\left(\alpha \right) \cdot R \cdot S\left(\alpha \right)$ on LP basis, where $S\left(\alpha \right)$ is a standard rotation matrix. This Jones matrix can be easily transformed to CP basis, given by the relation of $R^{{\mathrm{CP}}}\left(\alpha \right) = {{\Lambda }}^{ - 1} \cdot R\left(\alpha \right) \cdot {{\Lambda }}$, with $${{\Lambda }} = 1/\sqrt 2 \left[ {\begin{array}{*{20}{c}} 1 & 1 \\ { - j} & j \end{array}} \right]$$. Assuming that cross-LP condition $|r_{xy}| = |r_{yx}| = 1$ and $\varphi _{xy} = \varphi _{yx}$, we immediately achieve $$R^{{\mathrm{CP}}}\left(\alpha \right) = \left(\begin{array}{cc}r_{xy}e^{j\left({\varphi _{xy} - 2\alpha + \pi /2} \right)}& 0 \\ 0 & r_{xy}e^{j\left({\varphi _{xy} + 2\alpha - \pi /2} \right)}\end{array} \right) = \left(\begin{array}{cc}r_{\left| {\sigma _+ } \right.\rangle }e^{j\delta_{\left| {\sigma _+ } \right.\rangle }} & 0\\ 0 & r_{\left| {\sigma _- } \right.\rangle }e^{j\delta _{\left| {\sigma _- } \right.\rangle}}\end{array} \right)$$. This shows that involving both geometric ($e^{ - 2aj}$) and dynamic phase ($\varphi _{xy}$) completely decouples the copolarized component of $\delta _{\left| {\sigma _ + } \right\rangle }$ and $\delta _{\left| {\sigma_ - } \right\rangle }$. Moreover, it also indicates that cross-LP conversion properties of a meta-atom determine the efficiency of the entire cloak.

Theoretically, if we simultaneously impart two independent cloaking-phase patterns ($\delta _{\left| {\sigma _ + } \right\rangle }$ and $\delta _{\left| {\sigma _ - } \right\rangle }$), the cloak is expected to operate at any SOP in Poincare spheres. The underlying reason is that any incident polarization state can be described by the superposition of two opposite CP states, i.e., $\left| \sigma \right.\rangle = \chi _ + \left| {\sigma _ + } \right.\rangle + \chi _ - \left| {\sigma _ - } \right.\rangle$ with *χ*_+_ and *χ*_−_ representing the different proportionality coefficients. By comparing the above matrix $R^{{\mathrm{CP}}}\left(\alpha \right)$ on two sides, we can obtain two equations: $\delta _{\left| {\sigma _ + } \right\rangle } = \varphi _{xy} - 2\alpha + \pi /2$ and $\delta _{\left| {\sigma _ - } \right\rangle } = \varphi _{xy} + 2\alpha - \pi /2$. Then the required dynamic cross-LP-phase patterns $\varphi _{xy}$ and geometric-phase patterns $\varphi _g = 2\alpha$ to achieve simultaneous invisibility at $\left| {{\upsigma}_ + } \right\rangle$ and $\left| {{\upsigma}_ - } \right\rangle$ states are synthesized as

2a $$\varphi _{xy} = \varphi _{yx} = \frac{1}{2}\left({\delta _{\left| {\sigma_+ } \right\rangle } + \delta _{\left| {\sigma _- } \right\rangle }} \right)$$

2b $$\alpha = \frac{1}{4}\left({\delta _{\left| {\sigma _+ } \right\rangle } - \delta _{\left| {\sigma _- } \right\rangle } + \pi } \right)$$

### Design of full-polarization conformal-skin cloak

**Fig. 2:**
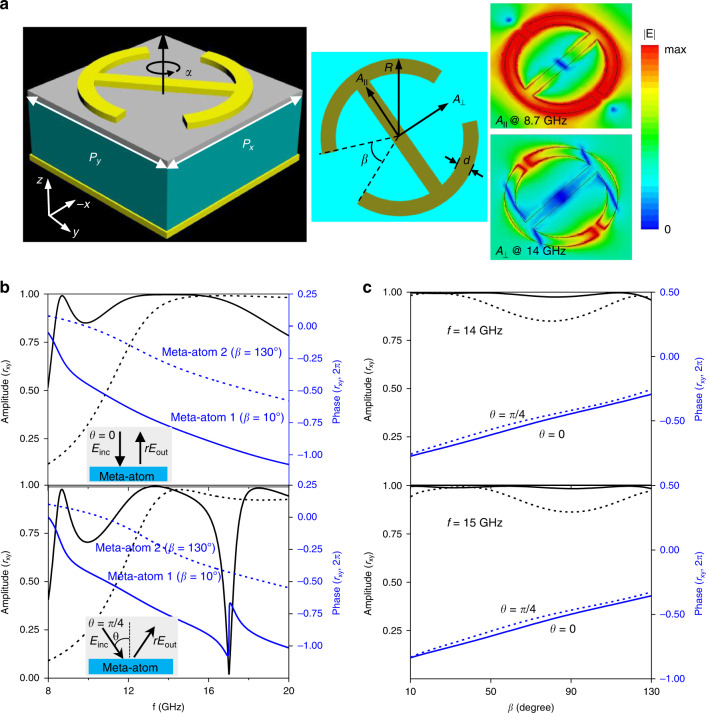
Design and characterization of the meta-atom for our metasurface cloak. **a** Layout of the reflection meta-atom with electric-field distributions corresponding to dual modes at 8.7 and 14 GHz shown in the inset. The anisotropic metal–insulator–metal meta-atom composed of an ABS-M30 plate sandwiched by top quasi-I-shaped metallic patterns oriented along *α* = 45° and bottom-flat ground. **b** Numerically calculated cross-LP reflection amplitude and phase spectrum for meta-atoms of *β* = 10° (solid line) and *β* = 130° (dashed line) under incidence angle of *θ* = 0 (top panel) and *θ* = π/4 (bottom panel). **c** Numerically calculated cross-LP reflection amplitude and phase versus *β* under *θ* = 0 and π/4 and frequency of 14 (top panel) and 15 GHz (bottom panel). The detailed geometric parameters are *p*_x_ = *p*_y_ = 5.5 mm, *d* = 0.4 mm, and *R* = 2.2 mm

Taking this general principle into consideration, an anisotropic building block is devised to realize |$r_{xy}$| = |$r_{yx}$| = 1 and $\varphi _{xy} = \varphi _{yx}$ and the aforementioned decoupled phase patterns in Eq. (2). We performed the full-wave numerical simulation of a meta-atom using the finite-difference time-domain (FDTD) method. As illustrated in Fig. 2a, the basic block (i.e., the meta-atom) utilized for our metasurface cloak can be produced using 3D-printing technique. It consists of an anisotropic metal–insulator–metal reflective meta-atom composed of an ABS-M30 plate sandwiched by top quasi-I-shaped metallic patterns and bottom-flat ground etched on two thin flexible substrate boards. We utilize the top circular I-shape resonator oriented along *α* = 45^o^ to break the symmetry along *x* and *y* axes and thus to generate two chirality-assisted characteristic modes ($A_\parallel$ and $A_ \bot$), with major electric-field distribution parallel and perpendicular to principal axis under SOP of $\left| {\sigma _x} \right\rangle$ and $\left| {\sigma _y} \right\rangle$, which can be evidenced by two cross-LP $r_{xy}$ peaks shown in Fig. 2b. These two interelement modes can be judiciously employed and cascaded to engineer a broadband high-efficiency cross-LP system. Moreover, we change the open-angle *β* to adjust the dynamic phase $\varphi _{xy}$. As shown in Fig. 2b and Supplementary Fig. S1, the meta-atom with α = 45° and *β* = 10° exhibits a broadband high cross-LP rate ($r_{xy}$ > 0.85) across 8.4–18.9 GHz (a fractional bandwidth of 77%) under *θ* = 0^o^. By changing *β* from 10 to 130°, a continuous phase change of $\varphi _{xy}$ with a maximum of 180° is achieved across the above entire band. To satisfy a full 2π phase coverage, an additional 180° phase jump is introduced by changing *α* by 90° without altering $r_{xy}$ significantly, see Supplementary Fig. S2. All the above results assist to construct the meta-atom library for the final cloak design. More importantly, the phase response against the incidence angle *θ* is quite close at 14 and 15 GHz as depicted in Fig. 2c, which exhibits a maximum phase tolerance of 15° and 20° when *θ* alters from 0 to 45°. Such a quasi-angle-independent phase response is particularly essential for a cloak with arbitrary boundaries, where the phase error induced by different incident angle *θ* can be minimized. Although slight fluctuation of $r_{xy}$ is observed at *θ* = 45°, it is still above 0.85 for all *β*, which should pose negligible effect in preserving the amplitude of our cloak. The physics of angle-insensitive EM response that lies in the 45° orientation considerably reduces the near-field coupling strength among adjacent meta-atoms (identified from the significantly low-field intensity at the edge of the meta-atom), which contributes mostly to the frequency shift^50^.

In the proof-of-concept demonstrations, we choose a trapezoid platform to initialize our full-polarization cloak design, while other geometries could also be implemented following our strategy. The metasurfaces are composed of a stacked composite of ABS-M30 and a thin F4B metallic ground and are characterized by the width *P*, and cross-section tilt angle ψ and length *L*. Given the parameters of the trapezoid bump, the theoretically required dual-phase patterns $\delta _{\left| {\sigma _ + } \right\rangle }$ and $\delta _{\left| {\sigma _ + } \right\rangle }$ and followed by the synthesized $\varphi _{xy}$ and *α* can be readily achieved. Finally, the layout of our conformal-skin cloak composed of spatially varying meta-atoms can be mapped by selecting meta-atoms from the library according to the target $\varphi _{xy}$ (*β*) and *α* distributions through a program code automatically performed CST Microwave Studio, see the CAD process in “Methods”. Two conformal-skin cloaks on 3D trapezoid and pyramid platform are reported with their invisibility property characterized in both near-field (NF) and far-field (FF) results under different excitation scenarios.

### Conformal-skin cloak on a 3D trapezoid platform

**Fig. 3:**
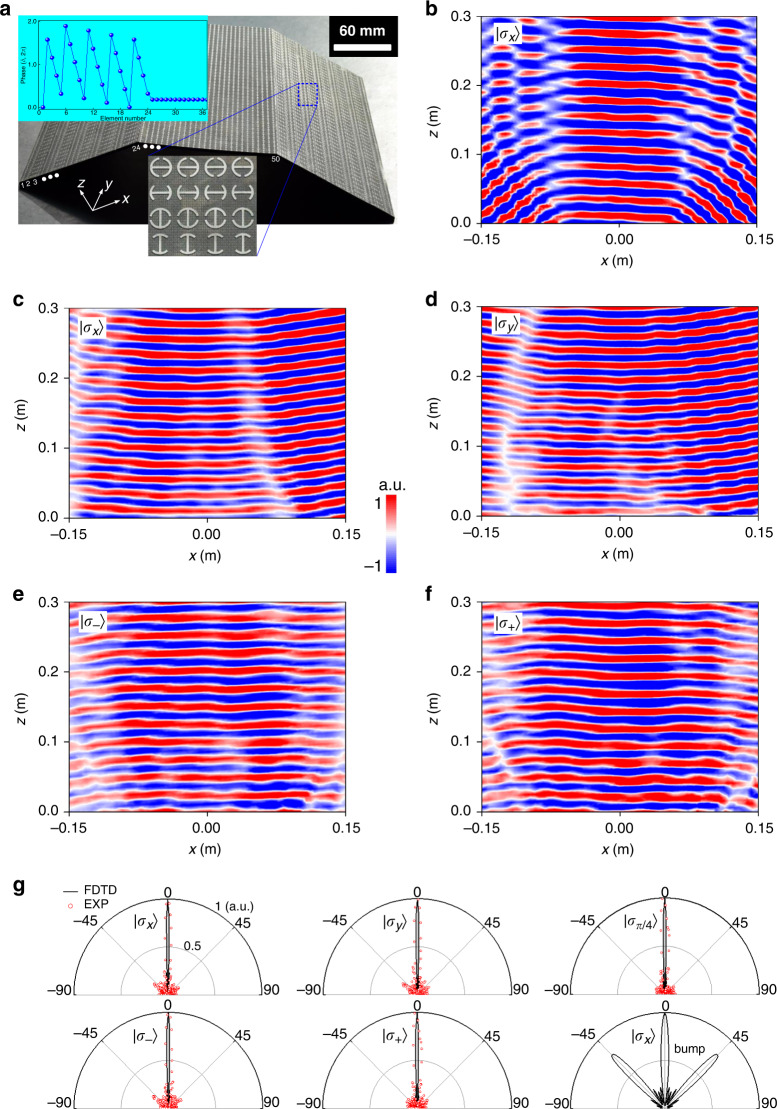
Characterization of the full-polarization trapezoid conformal-skin cloak under normal incidence. **a** Photograph of the fabricated sample with the magnified picture and phase profile along the centered *x* axis shown in the inset. Here, the phase profile is given only for half-cloak on *xz* plane since it is symmetric about the *x* axis for the left-half counterpart. The trapezoid bump is with a tilt angle of *ψ* = 22.5^o^ and a cross section of *L* × *H* = 387 mm × 50.5 mm. There are totally 24 meta-atoms on each slope, 26 meta-atoms on the top side along the *x* direction, while 40 meta-atoms along the *y* direction (*P* = 220 mm) of the cloak. **b** Measured NF *E*_x_ distributions of the bare bump under $\left| {\sigma _x} \right\rangle$ (**b**). **c**–**f** Measured NF *E*-field distributions (copolarized component) of cloaked bump under $\left| {\sigma _x} \right\rangle$ (**c**), $\left| {\sigma _y} \right\rangle$ (**d**), $\left| {\sigma _ - } \right\rangle$ (**e**), and $\left| {\sigma _ + } \right\rangle$ (**f**) at 15.5 GHz. **g** Comparison of copolarized FF scattering patterns on linear scale of 0–1 between FDTD simulations and experiments at 15 GHz on the *xz* plane for both cloaked bump under $\left| {\sigma _x} \right\rangle$, $\left| {\sigma _y} \right\rangle$, $\left| {\sigma _{\pi /4}} \right\rangle$, $\left| {\sigma _ - } \right\rangle$, and $\left| {\sigma _ + } \right\rangle$, respectively, and bare metallic bump of the same size under $\left| {\sigma _x} \right\rangle$. Here, all NF and FF results are normalized to their respective maximum with identical units and scales. The cloak sample is designed using a computer-aided design (CAD) process and prepared based on a four-step dual-sided fabrication process by combining 3D-printing and flexible printed circuit board (PCB) technique, see Sample preparing and fabrication in Methods. The commonly available 0.1-mm-thick F4B board with *ε*_r_ = 2.65 and tan*δ* = 0.001 is utilized as the flexible thin substrate and backed ground. By taking the stability and rigidity into consideration, the 2.5-mm-thick polymer ABS*-*M30 with *ε*_r_ = 2.7 and tan*δ* = 0.005 is chosen as the 3D-printing material to preserve the perfect shape of the supporting platform, and thus hold the well-designed phase profiles

We first design a conformal-skin cloak targeted at 15 GHz on a 3D trapezoid platform, which is characterized by *ψ* = 22.5°, top/bottom length *L*_1_ = 143/*L*_2_ = 387 mm, and height *H* = 50.5 mm in the triple-side cross section. Figure 3a and Supplementary Fig. S3 show the layout and parametric illustration of our designed cloak wrapped over a trapezoid metallic bump, according to the theoretically calculated phase profile shown in the inset of Fig. 3a. As indicated, our cloak is assembled by triple sub-metasurfaces with both spatially varied *β* and *α* for each meta-atom. The spatially varied *α* with *α* = 0 and 90^o^ plays a key role as it guarantees the CP polarization insensitivity. The underlying physics is that the phase response of the above specific meta-atoms with 0 and 90^o^ orientation under $\left| {\sigma _ + } \right\rangle$ and $\left| {\sigma _ - } \right\rangle$ state is the same in terms of equal values of *e*^i2*α*^ and *e*^−i2*α*^. Such feature guarantees to preserve both output copolarized phase and amplitude of all meta-atoms across the cloak for all SOP in terms of synergizing cross-LP dynamic phase through varying *β* and geometric phase by altering *α*, which distinguishes our design from any existing metasurface cloak^29–37^. In numerical and experimental characterizations, the bare and cloaked bump are normally illuminated by $\left| {\sigma _x} \right\rangle$, $\left| {\sigma _y} \right\rangle$, $\left| {\sigma _{\pi /4}} \right\rangle$, $\left| {\sigma _ + } \right\rangle$, and $\left| {\sigma _ - } \right\rangle$ plane wave incident on *xz* plane. Here, the polarization angle ($\phi$) of an LP light can be arbitrarily engineered by changing the azimuthal illumination angle/rotating the cloak about the vertical axis. Figure 3b–f plots the experimentally measured NF *E*-field patterns for both bare and cloaked bump at 15.5 GHz by scanning an area of 0.3 × 0.3 m^2^ on *xz* plane. All NF results are in good consistency with numerical simulations under $\left| {\sigma _x} \right\rangle$, $\left| {\sigma _y} \right\rangle$, $\left| {\sigma _ + } \right\rangle$, and $\left| {\sigma _ - } \right\rangle$ plane-wave illumination (Fig. 1c), except that the center operation frequency has slightly shifted from 15 to 15.5 GHz in the implementation, see NF patterns at other frequencies of a shifted bandwidth in Supplementary Section 3. As expected, Fig. 3b presents the distortion and splitting of light into various directions after reflecting from for a bare bump. In sharp contrast, the signal reflected from our metasurface-covered bump presents an almost flat and reconstructed wavefront with uniform intensity for all incident polarizations (Fig. 3c–f). With respect to the results presented in ref. ^29^, where the object is perfectly hidden for $\left| {\sigma _x} \right\rangle$ but completely visible by switching polarization, our approach works for both polarizations and exhibits a desirable operation bandwidth of 2.5/3 GHz (experiment/FDTD) within 14.5–17/14–17 GHz, corresponding to a fractional bandwidth of 16.7/20% (see Supplementary Figs. S4–S6 for more numerical and experimental NF and FF results at other frequencies). Such a level of bandwidth is very remarkable relative to the existing metasurface cloaks^29–37^.

The demonstrated mirror reflections indicated by NF results can be further verified from the highly directive single-mode FF specular scattering patterns shown in Fig. 3g, where an excellent agreement is observed between numerical calculation and experimental data. The slightly larger fluctuations of sidelobes and wider width of the main beam in the latter case are attributed to the nonideal plane -wave excitation and insufficient directivity of the horn excitation antenna. Nevertheless, when the metasurface cloak is removed, both NF and FF results turn out to be substantially distorted with triple-scattering modes directing backward, −45° and 45°, revealing a mirror function of three sides of the trapezoid bare bump. The sharp scattered specular beam with other scattering modes at oblique angles completely suppressed indicates high invisible performance.

### Conformal-skin cloak on a 3D pyramid platform

**Fig. 4:**
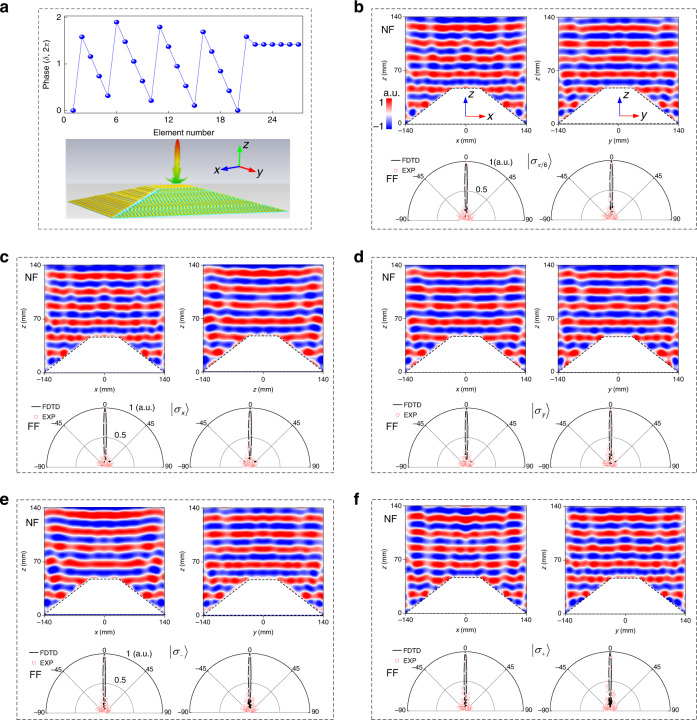
Characterization of the full-polarization pyramid conformal-skin cloak at 15 GHz under normal incidence. **a** Phase profile for half-cloak along centered *x* or *y* axis, i.e., 21 meta-atoms on each slope and 6 meta-atoms on the top; the inset shows FDTD-calculated 3D FF patterns in linear scale from the actual metasurface cloak for a quick view of mirror backward scattering. Here, the phase profile is given only for a half cross section of the *xz/yz* plane since it is symmetric about the centered *x* and *y* axes. **b**–**f** FDTD- calculated NF copolarized *E*-field distributions (top panel) and comparison of copolarized FF scattering patterns (bottom panel) between FDTD simulations and experiments in both *xz* (left panel) and *yz* (right panel) planes for cloaked bump under polarization states of $\left| {\sigma _{\pi /6}} \right\rangle \,({\mathbf{b}})$, $\left| {\sigma _x} \right\rangle \,({\mathbf{c}})$, $\left| {\sigma _y} \right\rangle \,({\mathbf{d}})$, $\left| {\sigma _ - } \right\rangle$ (**e**), and $\left| {\sigma _ + } \right\rangle \,({\mathbf{f}})$. The trapezoid bump is with a tilt angle of *ψ* = 22.5^o^. The height of the pyramid is *H* = 44.5 mm, while the length four top and four bottom sides are *L* = 66 mm and *L* = 280.9 mm, respectively. There are a total of 144 meta-atoms while 635 meta-atoms within the top face and each of four-side face. The phase difference caused by different thickness of the ABS-M30 between the top (*h* = 2.5 mm) and side (*h* = 2.3 mm) faces is compensated by 19^o^ at 15 GHz in the initial design

**Fig. 5:**
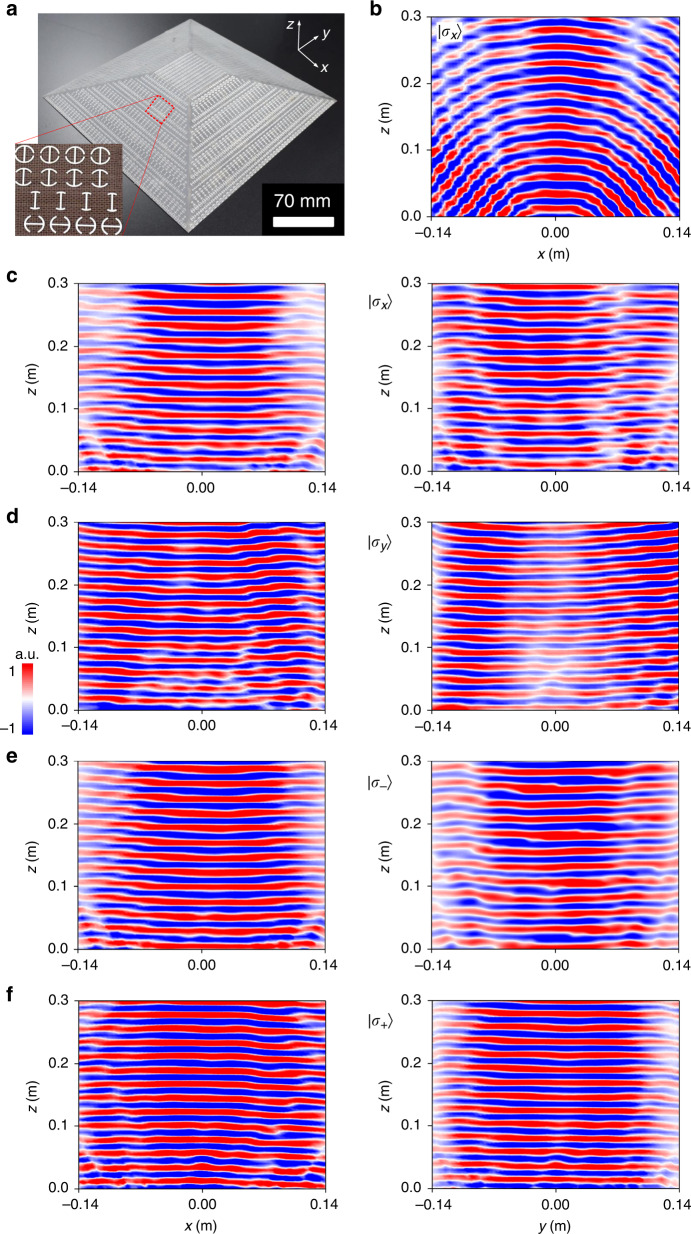
Experimental characterization of the pyramid bump and full-polarization conformal-skin cloak at 15.5 GHz under normal incidence. **a** Photograph of the fabricated sample with the magnified picture shown in the inset. **b**–**f** NF copolarized *E*-field distributions of the bump under $\left| {\sigma _x} \right\rangle \,({\mathbf{b}})$ and (**c**–**f**) metasurface cloak under $\left| {\sigma _x} \right\rangle$ (**c**), $\left| {\sigma _y} \right\rangle$ (**d**), $\left| {\sigma _ - } \right\rangle$ (**e**), and $\left| {\sigma _ + } \right\rangle \,({\mathbf{f}})$ wave in *xz* (left panel) and *yz* plane (right panel)

The above full-polarization conformal-skin cloak on a 3D trapezoid platform can be further extended to a 3D pyramid platform with the full-azimuthal plane operation. For this purpose, we assembled a cloak composed of five sub-metasurfaces on a square pyramid with *ψ* = 22.5°, *H* = 44.5 mm, and *L*_1_ = 66/*L*_2_ = 280.9 mm according to the theoretically calculated phase patterns $\delta _{\left| {\sigma _ + } \right\rangle }$ = $\delta _{\left| {\sigma _ - } \right\rangle }$, see Fig. 4a, Fig. 5a, and Supplementary Fig. S7. The theoretically calculated phase profile is symmetric with respect to the center *x* and *y* axes. All meta-atoms disposed on the top- and side- tilted faces are constructed automatically in CST Microwave Studio relying on a rigorous calculation of spatial coordinates at each position. Since both dynamic and geometric phases are involved, the meta-atoms in two *x*-orientated tilt faces should be deliberately arranged orthogonally, point by point, to those in two *y*-orientated tilt faces aiming to compensate the phase difference induced by spatial variation. To comprehensively evaluate the performances, NF and FF results are recorded in both principal *xz* and *yz* planes under five representative polarization states of normally incident plane EM waves. Elegant cloaking performance can be also expected at other azimuthal planes, except for the above two principal planes.

As shown in Fig. 4b–f, we observe desirable flat wavefronts reflected in both planes with almost uniform strength after impinging on the cloak under all inspected SOP. Minor distortions of NF patterns, especially for the unexpected interference, are partially attributed to the nonideal infinite boundary in 3D simulation, which takes lot of computation resources in calculating a large-volume cloak. They are partially induced by the nonuniform scattering amplitudes of those meta-atoms at top and four-side surfaces since they are considered to be positioned at different angles with respect to the given illumination. It can be explained in Fig. 2c that the scattering amplitudes will slightly vary under different incident angles. Fortunately, such minimal fluctuation does not pose many penalties on the invisibility, which can be further verified from FF scattering patterns. Well-preserved specular reflections are observed for all SOP and planes. As shown in Fig. 5b, the fan-shape wavefront is clearly observed for the bare bump. However, it is completely flattened on both planes when wrapping the bump with our conformal-skin metasurface cloak (Fig. 5c–f). More importantly, such desirable wavefront is achieved under four representative polarization states of $\left| {\sigma _x} \right\rangle$, $\left| {\sigma _y} \right\rangle$, $\left| {\sigma _ + } \right\rangle$, and $\left| {\sigma _ - } \right\rangle$. The cloaking performances at other frequencies are also numerically and experimentally evaluated, see Supplementary Figs. S8, S9 for numerical NF patterns, Figs. S10–S13 for measured NF patterns, and Fig. S14 for comparison of far-field scattering patterns between numerical calculations and experimental measurements. The pyramid cloak exhibits almost the same working bandwidth (~20%) relative to its trapezoid counterpart, but circumvents the formidable issue of full-azimuthal operation.

Finally, we also evaluated the cloaking performance of our pyramid cloak at oblique incidence by illuminating the sample with light incident along *θ* = −20° and *θ* = −30° on *xz* and *yz* plane. As shown in Supplementary Fig. S15, the pyramid cloak preserves its mirror-like far-field scattering patterns for *θ* = −20° and *θ* = −30° at four representative frequencies with negligible sidelobes. Degraded cloaking performances occur at large *θ*, in terms of large sidelobes, wider beam, and reduced bandwidth. This is especially true for the FF scattering pattern obtained at 14.5 GHz and θ = −30°. It should be strengthened that the cloaking performance is inevitably weakened for oblique incidence and off-*f*_0_ operation since the cloak was originally designed at *f*_0_ for normal incidence according to Eq. (1). Nevertheless, due to the angle-immune and broadband scattering intensity and phase of meta-atoms with alternatively changed *α* = 0° and 90° (small interelement coupling) and the small height of the cloak, all results indicate an elegant angle-adaptive cloaking behavior within a bandwidth of 2.5 and 2 GHz for *θ* = −20° and *θ* = −30°, respectively. However, for larger incident angles, the cloaking performance would be sharply exasperated due to the magnified phase tolerance and a renewed specific design is necessary. More importantly, our pyramid cloak indeed enables a real 3D operation (full-azimuth operation) under arbitrary azimuthal-angle nonsymmetric illumination, see Supplementary Fig. S16, where again similar mirror reflection is clearly inspected under two scenarios of EM wave excitation with $\phi = 30$° and *θ* = 10°, and $\phi = 40$° and *θ* = 20°. Since our conformal-skin cloak is ultrathin, the lateral shift of the reflected beams observed in bulk cloaks is automatically diminished.

## Discussion

We have proposed and experimentally verified a deterministic strategy of invisible cloaking via a conformal-skin metasurface cloak for full polarization described by Poincaré spheres. The challenging polarization-independent operation is theoretically guaranteed by imposing two sets of cloaking phases on two decoupled orthogonal spins. We have devised two sophisticated trapezoid conformal-skin cloaks combining 3D printing and flexible PCB technique. Our proposed method is capable of preserving predesigned scattering signatures (amplitude and phase) across an elegant bandwidth under fully polarized light. Moreover, the constraints of impedance mismatching and lateral shift of reflected beams in existing invisibility cloaks are also mitigated. In principle, there is no cloaking shape and size limitation endowed by the 3D-printing fabrication. Although the height of our designed prototype cloak is 2.5 *λ*_0_ tall, further FDTD results indicate that it can be considerably increased provided that the slope angle of the platform *ψ* < 90°, posing no theoretical limitation on the size of the cloaking region. Furthermore, we have designed two metasurface cloaks in both cross-LP and co-LP systems based on the available dynamic-phase approach, see Supplementary Figs. S17 and S18. The results unanimously confirm that these cloaks cannot preserve polarizations upon either LP- or CP-state operations. Table 1 summarizes the feature of some of the experimentally reported metasurface cloaks. To the best of our knowledge, our metasurface cloak with ultrathin thickness of only ~*λ*_0_/200, the bandwidth of 20%, and full-azimuth operation, presents the best performance among available passive cloaks. Our paradigm opens up an unprecedented avenue to ultrathin and robust cloaking of arbitrary-shape 3D objects, advancing a meaningful step toward realistic applications.

**Table 1:** Summary of previously reported metasurface cloaks

References	Azimuth operation	Polarization	*λ*_0_	Thickness	Bandwidth	Method
Ni et al.^29^	Specific	Single LP	730 nm	*λ*_0_/10	Not reported	Nano patch (Co-LP)
Yang et al.^30^	Full	Two LPs	37.5 mm	*λ*_0_/20	18.2%	Square ring (Co-LP)
Orazbayev et al.^31^	Specific	Two LPs	3.75 mm	*λ*_0_/19	8%	Circular ring (Co-LP)
Jiang et al.^32^	Specific	Two LPs	46.8 mm	*λ*_0_/17	~7.4%	Circular ring (Co-LP)
Huang et al.^36^	Specific	Single LP	125 mm	*λ*_0_/26	4.2%	Semicircle patch (Co-LP)
Qian et al.^37^	Specific	Single LP	37.5 mm	*λ*_0_/18	~31%	Back-to-back semi-ring (Co-LP)
This work	Full	Insensitive	20 mm	*λ*_0_/200	20%	Diagonal I resonator (Cross-LP)

## Materials and methods

### Numerical characterizations

All numerical designs and FDTD characterizations are performed through the numerical simulation package CST Microwave Studio. Specifically, in calculations of the reflection amplitudes/phases of the meta-atom, especially in generating the reflection response database, we impose periodic boundary conditions at its four bounds, and with a Floquet port placed at a distance 15 mm away from the meta-atom plane in the frequency-domain solver of the commercial software. In NF and FF numerical characterizations of the polarization-sensitive and full-polarization triangle, trapezoid, and pyramid cloaks, metasurfaces composed of 30 × 1 and 48 × 1 spatially varied meta-atoms along two slopes are utilized in time-domain solver with periodic boundary condition assigned to *y* sides to reduce the calculation volume, while four open boundaries set along *x* and *z* axes. In full-wave FDTD evaluations of the cloaking performance, all boundaries of the square pyramid are arranged as open conditions.

### Sample preparing and fabrication

The cloak sample is prepared based on a four-step dual-sided fabrication process by combining 3D-printing and PCB technique. The supporting trapezoid platform with specific tilt angle is prepared using 2.5-mm-thick 3D-printing polymer material ABS-M30 (dielectric constant *ε*_r_ = 2.7 and loss tangent tan*δ* = 0.005) through 3D-printing technique, see Supplementary Fig. S17. The top and bottom metallic patterns and ground of our metasurface cloak are fabricated individually on two 0.1-mm-thick flexible F4B dielectric boards (*ε*_r_ = 2.65 and tan*δ* = 0.001) using the PCB technique. A CAD process is established, which can automatically construct all metallic patterns through program codes in a commercial software based on reflection response and position database of each meta-atom. After all PCB boards and supporting platforms are ready, the next step is to align and attach each flexible board to two sides of the ABS-M30 platform to form an entirety through adhesives. Finally, they are shaped and reinforced by clamps for several hours. Such an assembling process avoids metalizing the 3D-printed substrates through thin-film sputtering.

### Microwave experiments

All far-field (FF) and near-field (NF) experiments are performed in a microwave anechoic chamber to avoid possible interference from the environment, see the experimental setup shown in Supplementary Fig. S18. Two pairs of highly directive LP or CP antenna emitting Gaussian wave are utilized as receiver and transmitter. The double-ridged horn exhibiting a voltage standing-wave ratio (VSWR) less than 2.5 within the frequency range 1–18 GHz is utilized as the LP antenna. By altering the orientation of the emitting antenna with respect to the fixed sample, a LP wave excitation can be readily realized with several representative polarization angles of 0, 30, 45, and 90^o^. For CP wave excitation, the sample is illuminated by a horn with an axial ratio of less than 3.5 dB, and a voltage standing-wave ratio of less than 2.5 within 8–18 GHz.

In all NF contour maps, a 10-/15-mm-long monopole, functioning as the receiver, is placed between the 1-m-distanced sample and horn, and is connected to an AV3672B Agilent vector network analyzer to record the static EM signals. It is fixed to a 2D electronic step motor that can move automatically in a maximum area of 1.2 × 1.2 m with a step resolution of 5 mm. To guarantee the pure scattering signature, the incident signal in free space is deducted from the total fields. In the FF scattering pattern measurements, the cloak sample and the receive horn to record signals are fixed on a large rigid foam that is capable of rotating freely along the foam’s axial center. The transmitting horn is placed 6 m away to afford desired excitations.

## Supplementary information

SUPPLEMENTARY INFORMATION
