# Supplementary material for: Polarization-insensitive 3D conformal-skin metasurface cloak
Source: Light Sci Appl. 2021 Apr 8;10:75. doi: 10.1038/s41377-021-00507-8 (PMC8032745; doi:10.1038/s41377-021-00507-8)
Supplement: Supplementary file 1 — SUPPLEMENTARY INFORMATION [file 41377_2021_507_MOESM1_ESM.docx]

Supplementary Information for

**Polarization-Insensitive Conformal-Skin Metasurface Cloak**

*He-Xiu Xu^1,2^,* ^†^*,* ^*^*, Guangwei Hu^3^*^†^*, Yanzhao Wang^1^*^†^*, Chaohui Wang^1^, Mingzhao Wang^1^, Shaojie Wang^1^, Yongjun Huang^4^, Patrice Genevet^5*^, Wei Huang^2*^, Cheng-Wei Qiu^3*^*

*^1^Air and Missile Defense College, Air Force Engineering University, Xi'an 710051, China*

*^2^Institute of Flexible Electronics, Northwestern Polytechnical University, Xi'an 710072, China*

*^3^Department of Electrical and Computer Engineering, National University of Singapore, Singapore 117583, Singapore*

*^4^School of Information and Communication Engineering, University of Electronic Science and Technology of China, Chengdu 611731, China*

*^5^Université Côte d’Azur, CNRS, Centre de Recherche sur l’Hétéro-Epitaxie et ses Applications (CRHEA), 06560 Valbonne, France*

^†^These authors contribute equally to this work

*Corresponding Authors: He-Xiu Xu (hxxuellen@gmail.com); Patrice Genevet (pg@crhea.cnrs.fr); Wei Huang (iamwhuang@nwpu.edu.cn); Cheng-Wei Qiu (eleqc@nus.edu.sg)

**Keywords:** Polarization-insensitive; metasurface cloak; spin decoupling; phase-amplitude restoring; full-azimuth

1. **Generalized theory for the full-polarization conformal-skin cloak**

In the following, we generalize the theory to decouple phases at $\left| \left. \sigma_{+} \right\rangle\right.$ and $\left| \left. \sigma_{-} \right\rangle\right.$ states for both co-LP and cross-LP system, aiming to afford the basic design criterion for full-polarization invisibility in reflection geometry. Suppose an arbitrary meta-atom rotating an angle of *α* with respect to its central axis under the Cartesian coordinate system, then the emerging linear reflection complex Jones matrix $R^{LP}\left(\alpha\right)$ can be formulated as a function of its previous counterpart as $R^{LP}\left(\alpha\right)=S^{-1}(\alpha)\times R\times S(\alpha)$, where $S(\alpha)\text{=}\left(\begin{matrix} \cos\alpha& -\sin\alpha\\ \sin\alpha& \cos\alpha\end{matrix}\right)$. However, such a Jones matrix is derived as $R^{CP}\left(\alpha\right)=\Lambda^{-1}\bullet R^{LP}\left(\alpha\right)\bullet\Lambda$ in circular polarization (CP) basis under $|+\rangle$ and $|-\rangle$ state, where $\Lambda=\frac{1}{\sqrt{2}}\left(\begin{matrix} 1 & 1 \\ -j & j \end{matrix}\right)$. The exotic feature of an arbitrary meta-atom is indicated by a general matrix  , where , , , and are phase and amplitude spectrum of four $\left| \left. \sigma_{x} \right\rangle\right.$/$\left| \left. \sigma_{y} \right\rangle\right.$-polarized reflection components under excitation of $\left| \left. \sigma_{x} \right\rangle\right.$ and $\left| \left. \sigma_{y} \right\rangle\right.$ LP wave, respectively. These phases are corresponding to dynamic or propagation phases associated with structure parameters. For a reciprocal system without complete rotational and mirror symmetry breaking discussed here, we have |$r_{xy}$|=|$r_{yx}$| and ${\varphi_{xy}=\varphi}_{yx}$. Then, the reflection Jones matrix in CP basis can be formulated as

(1)

Eq. (1) reveals that the involving of both geometric phase ($e^{-2\alpha j}$) and propagation phase ($\varphi_{yx}$) enables completely decoupled $\delta_{\left| \left. \sigma_{+} \right\rangle\right.}$ and $\delta_{\left| \left. \sigma_{-} \right\rangle\right.}$. Noted that above diagonal and off-diagonal components in CP matrix should be reversed considering the fact of interchanged handedness in reflection.

In a complete co-LP system with mirror symmetry along x and y axis, there is no cross polarization ($r_{yx}\approx r_{xy}\approx0$) and we can easily engineer $r_{xx}\approx r_{yy}\approx1$. Moreover, the term carrying geometric phase should be unity while the residual $r_{++}$ and $r_{--}$ ought to approach zero in order to facilitate a high spin-conversion efficiency which is dependent on co-polarization rate. Taking these aspects into consideration, we impose and further simplify Eq. 1 as . By a comparison of the matrix on two sides, we immediately obtain two equations: and . In this case, we deduce the required propagation and geometric phase profiles to cloak objects under dual spin states.

(2a)

(2b)

$\alpha=\frac{1}{4}(\delta_{|\sigma_{+}\rangle}-\delta_{|\sigma_{-}\rangle})$ (2c)

On the contrary, for a complete cross-LP system without mirror symmetry, i.e., $r_{xx}\approx r_{yy}\approx0$, we immediately obtain and conclude that spin-decoupling efficiency is determined by the cross-LP rate. By a comparison of the matrix on two sides, we immediately obtain two equations: and . Then, the required LP phase patterns to achieve simultaneous invisibility at $\left| \left. \sigma_{+} \right\rangle\right.$ and $\left| \left. \sigma_{-} \right\rangle\right.$ states are synthesized as

(3a)

$\alpha=\frac{1}{4}(\delta_{|\sigma_{+}\rangle}-\delta_{|\sigma_{-}\rangle}+\pi)$ (3b)

1. **High-efficiency and broad bandwidth of the meta-atom**

In Fig. 2 of the main text, we have shown the high-efficiency and 180^o^ phase control at two typical *β* and two representative frequencies. Here, we further afford the full amplitude and phase spectrum at all scanned frequencies and *β* under two cases of *θ*=0 and *θ*=π/4, see Fig. S1. As is much appreciated, the near-unity high efficiency with continuous phase control by changing *β* from 10^o^ to 130^o^ is observed in both cases across a broad operation bandwidth. Such a level of broad bandwidth and high reflection rate is very beneficial for the design of frequency-insensitive cloak with preserved amplitude.

**
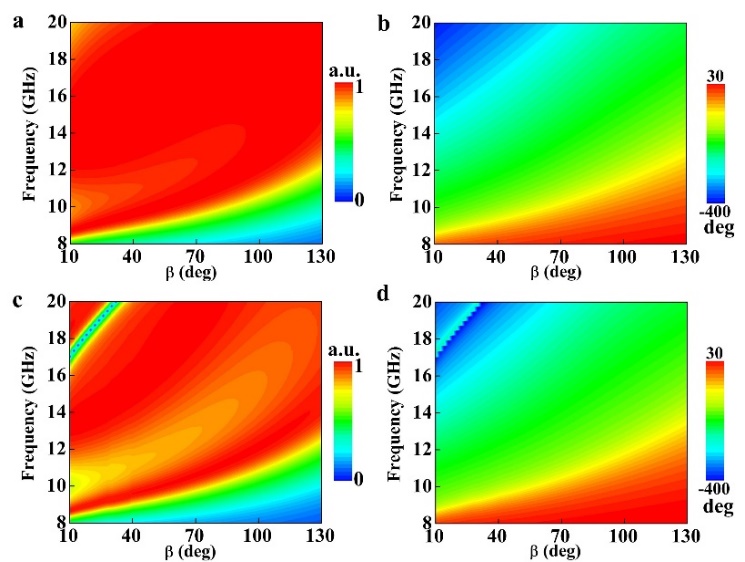
**

**Fig. S1** FDTD calculated cross-LP reflection (a, c) amplitude (|$r_{xy}$|) and (b, d) phase ($\varphi_{xy}$) of a basic meta-atom versus frequency and the parameter *β* under (a, b) normal (*θ*=0) and (c, d) oblique (*θ*=π/4) illumination.

Fig. S2 gives the FDTD calculated EM response of four meta-atoms with extreme value of *β*=10º and *β*=130º in constructing the metasurface cloak. As is clearly observed, the rotation of the meta-atom from α=45º to α=135º does not pose any effect to the reflection amplitude spectrum but induces an additional 180º phase delay in the phase response. Such a declaration still holds for the meta-atoms with *β* varying between 10º~130º. This is the key point for the full 2π phase cover which is necessary for the spin decoupling and metasurface design.


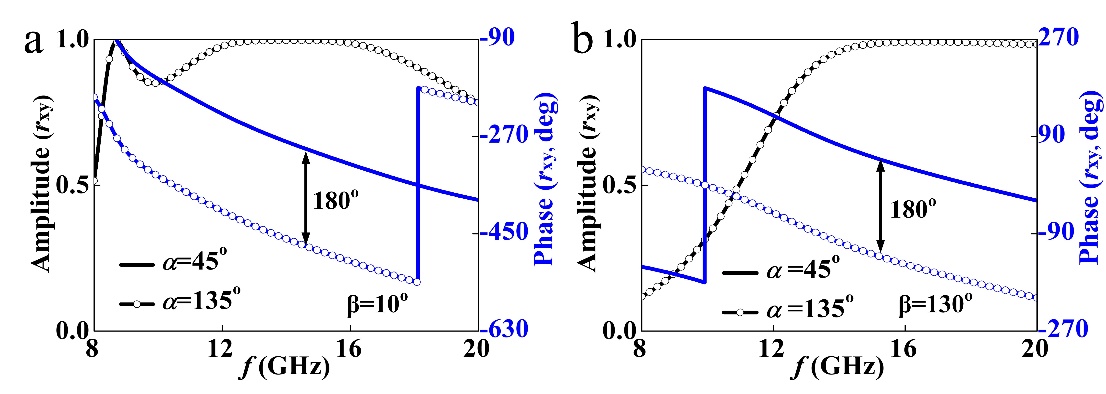


**Fig. S2** FDTD calculated cross-LP reflection amplitude (|$r_{xy}$|) and phase ($\varphi_{xy}$) spectrum versus frequency for the meta-atoms with (a) *β*=10º, (b) *β*=130º and α=45º (solid line), α=135º (circle symbol).

1. **Bandwidth of the trapezoid conformal-skin cloak**

Fig. S3 depicts the layout of the trapezoid metasurface cloak which is characterized by *ψ*=22.5^o^, top/bottom length *L*_1_=143/*L*_2_=387 mm and height *H*=50.5 mm in the triple-side cross section.


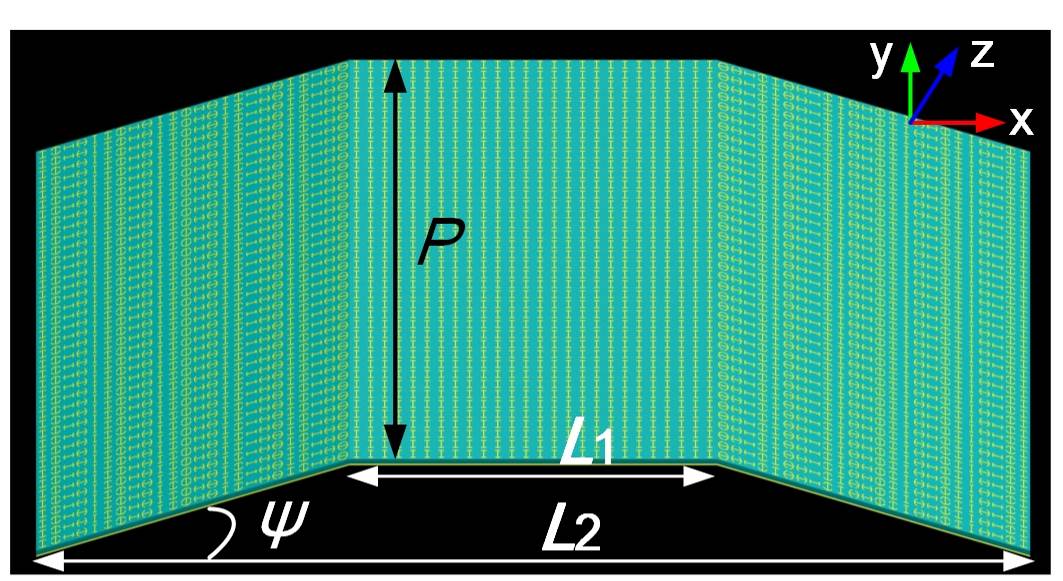


**Fig. S3** Layout and parametric illustration of the trapezoid conformal-skin cloak. The phase difference caused by different thickness of the ABS-M30 between the top face (*h*=2.7 mm) and four side faces (*h*=2.5 mm) is compensated by 13^o^ at 15 GHz in the initial design.

In Fig. 4 and Fig. 5 of the main text, we have discussed the invisible performance of the full-polarization trapezoid cloak at the center frequency. Here, we further evaluate the cloaking performance at other frequencies, aiming to illustrate the operation bandwidth. As can be seen from Fig. S4 and Fig. S5, reasonable agreement of NF results is observed at several representative frequencies of 14.5~17 GHz, where flat wavefront is clearly inspected with near uniform intensity. The experimental/FDTD operation bandwidth is about 2.5/3 GHz within 14.5~17/14~17 GHz, corresponding to a fractional bandwidth of 16.7/20%. Such a proposal can be further evidenced from the FF patterns shown in Fig. S6, where the single-mode backward scattering is also achieved within 14~17 GHz. Both NF and FF results reveal a mirror reflection behavior mimicking a flat ground. Such behavior does not deteriorate until at the low and upper edge frequencies of 14 and 17 GHz, where locally realigned wavefront occurring in NF patterns and large sidelobes and fluctuations appearing in the broadside widen FF beam. Beyond the edge frequencies, the object will be detectable again from the background. The phase error induced by the fixed theoretical phase profile and dispersive meta-atom gives rise to the deteriorative off-frequency cloaking behavior.


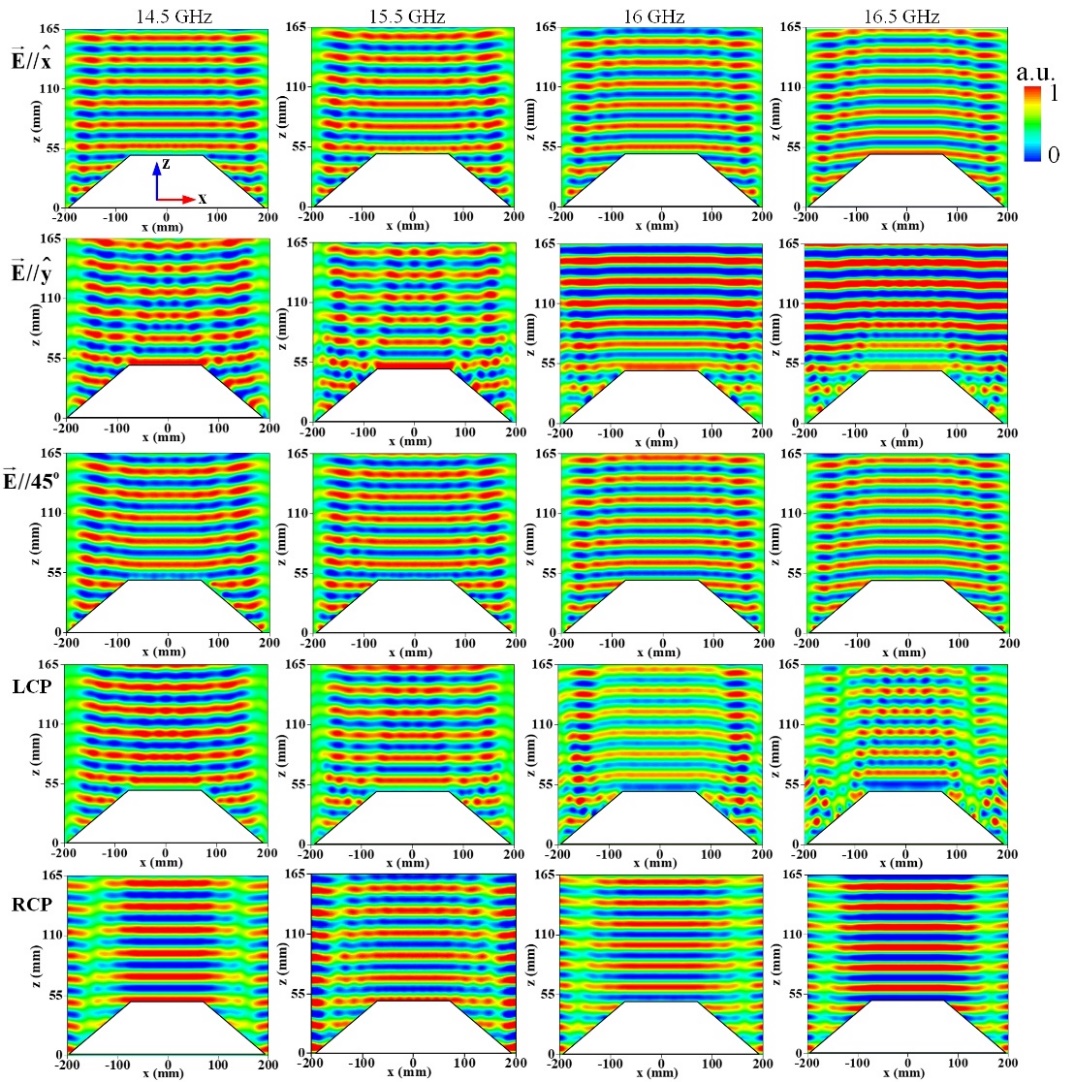


**Fig. S4** FDTD calculated NF co-polarized *E*-field distributions in xz cross-section plane of the trapezoid conformal-skin cloak at different frequencies of 14.5, 15.5, 16 and 16.5 GHz under normal incidence with $\overrightarrow{\text{E}}\text{//}\widehat{\text{x}}$, $\overrightarrow{\text{E}}\text{//}\widehat{\text{y}}$, $\overrightarrow{\text{E}}\text{//30}^{\text{o}}$, LCP and RCP wave.


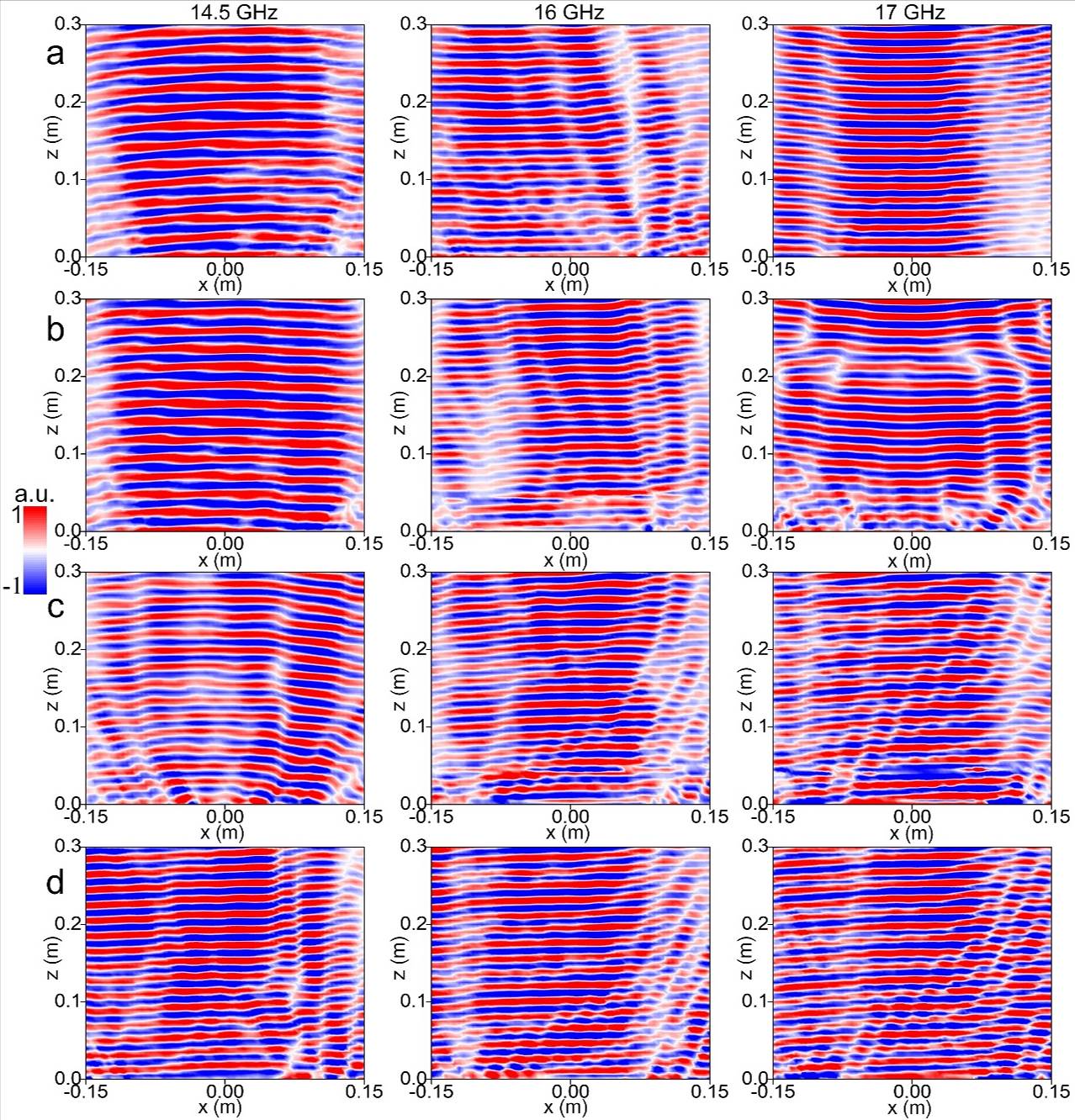


**Fig. S5** Experimentally measured NF co-polarized *E*-field distributions in xz cross-section plane of the trapezoid conformal-skin cloak at different frequencies of 14.5, 16 and 17 GHz under normal incidence with (a) $\overrightarrow{\text{E}}\text{//}\widehat{\text{x}}$, (b) $\overrightarrow{\text{E}}\text{//}\widehat{\text{y}}$, (c) LCP and (d) RCP wave.

**
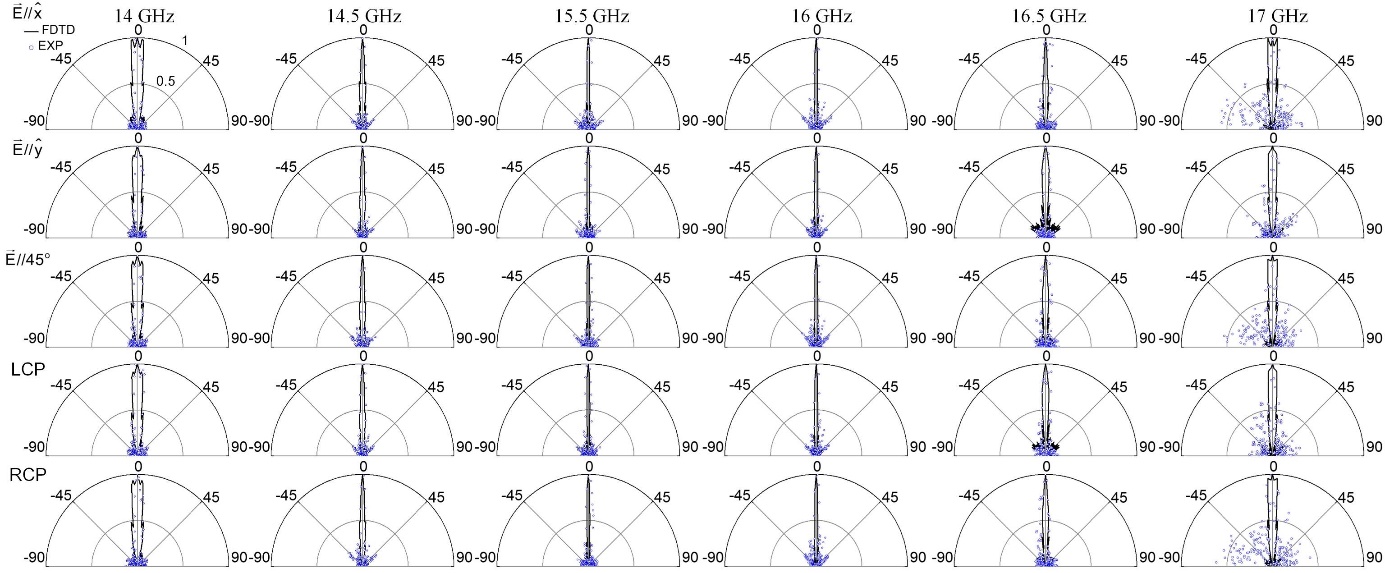
Fig. S6** FDTD calculated and experimentally measured co-polarized FF scattering patterns in xz plane of the trapezoid conformal-skin cloak at different frequencies of 14~17 GHz in steps of 0.5 GHz under normal incidence of different polarization states.

1. **Layout and bandwidth of the pyramid conformal-skin cloak**

**Fig. S7** portrays the complete layout of the full-polarization pyramid cloak for close inspection. All meta-atoms on top and sided tilt faces are constructed based on a rigorous control program performed automatically in a mathematical software (see CAD modeling process in Methods).


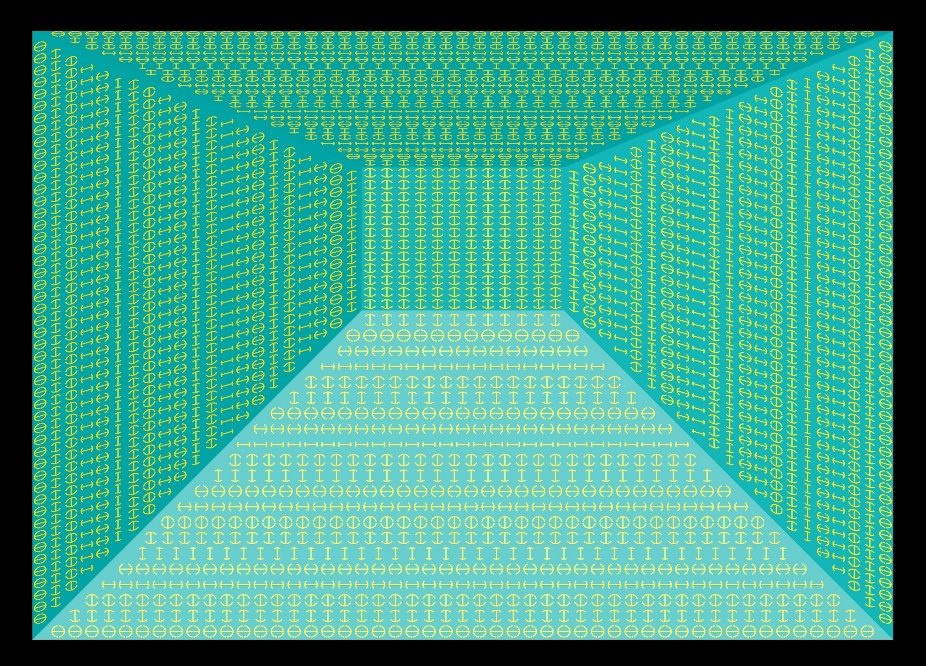


**Fig. S7** Layout of the pyramid conformal-skin cloak.

To further illustrate the working bandwidth of our pyramid cloak, we also evaluate the NF and FF patterns in both xz and yz planes at several representative off-center frequencies within 14~17 GHz under five representative polarization states, see Figs. S8 and S9 for FDTD NF patterns, Figs. S10~S13 for measured NF patterns, and Fig. S14 for comparison of FF scattering patterns between FDTD calculations and experimental measurements. As is shown in Figs. S8~S13, the fan-shape wavefront of the bare bump is flattened to some extent at all observed frequencies and polarization states. Slight fluctuations of fields are inspected across specific positions at some frequencies due to the non-uniform reflection amplitude of spatially varied meta-atoms. Nevertheless, as discussed in the main text such a level of distortion does not pose much penalty on the eventual cloaking performance. Such a proposal finds strong support from the FF scattering patterns depicted in Fig. S14. As is much appreciated, we inspected uniform pencil beams at all observed frequencies within 14~17 GHz. They are almost the same in most cases except for the larger sidelobe and beam splitting occurring at edge frequencies. The reason for the degenerated invisibility at off-target frequency is the same as that discussed in the main text. The pyramid metasurface cloak also exhibits an elegant operation bandwidth of 2.5/3 GHz (experiment/FDTD) within 14.5~17/14~17 GHz, corresponding to a fractional bandwidth of 16.7/20%.


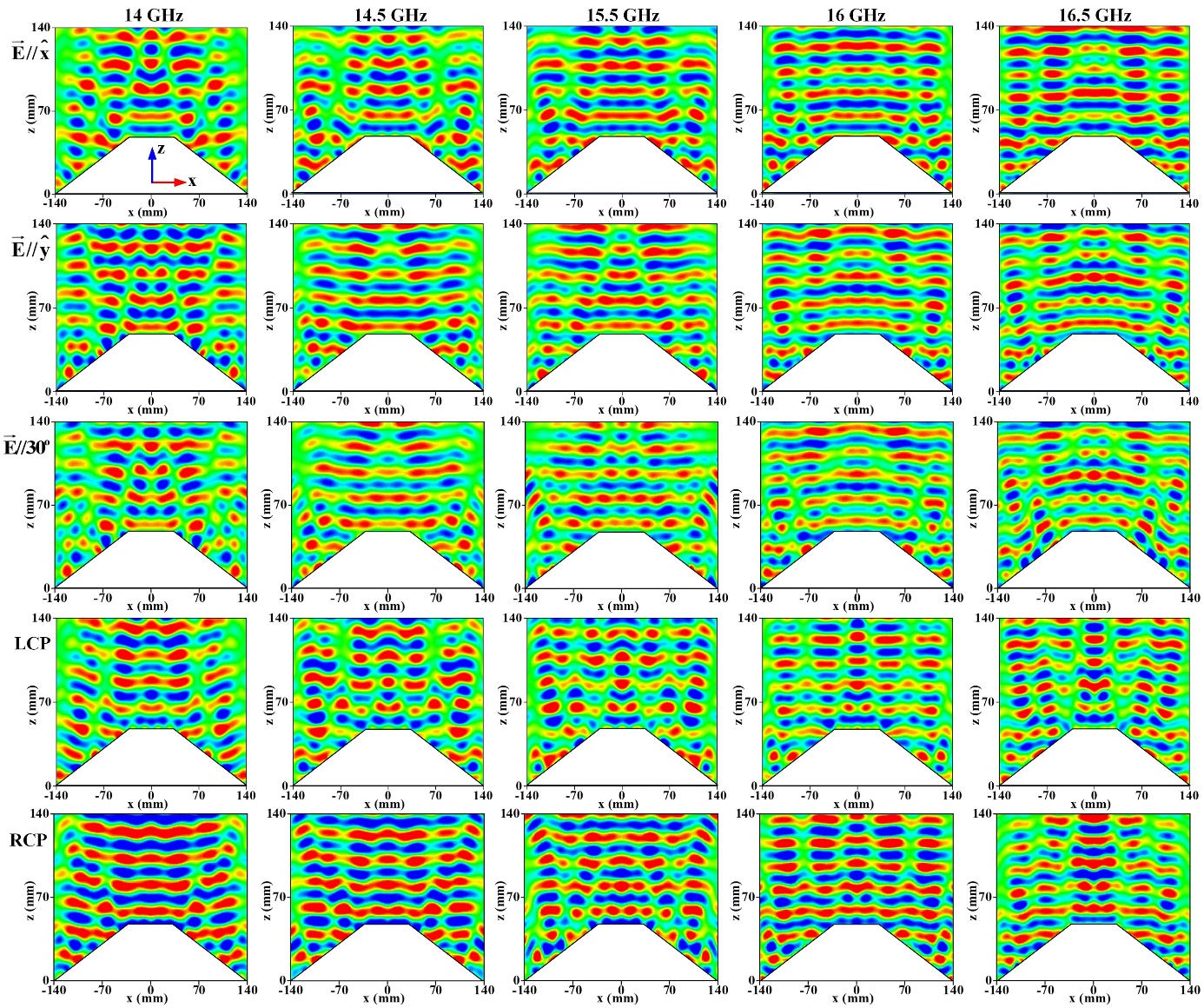


**Fig. S8** FDTD calculated NF co-polarized distributions in xz cross-section plane of the pyramid conformal-skin cloak at different frequencies of 14~16.5 GHz in steps of 0.5 GHz under normal incidence with $\overrightarrow{\text{E}}\text{//}\widehat{\text{x}}$, $\overrightarrow{\text{E}}\text{//}\widehat{\text{y}}$, $\overrightarrow{\text{E}}\text{//30}^{\text{o}}$, LCP and RCP wave.


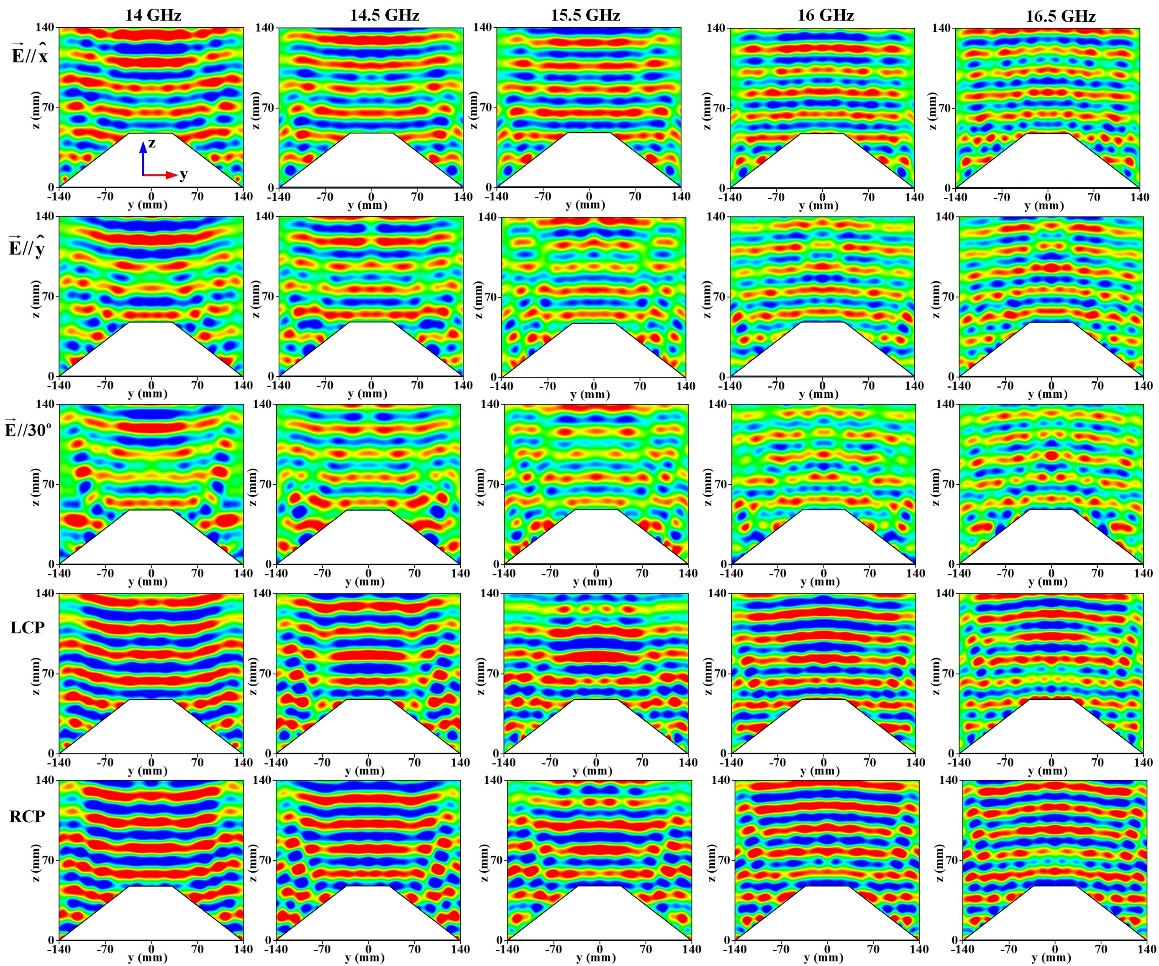


**Fig. S9** FDTD calculated NF co-polarized *E*-field distributions in yz cross-section plane of the pyramid conformal-skin cloak at different frequencies of 14~16.5 GHz in steps of 0.5 GHz under normal incidence with $\overrightarrow{\text{E}}\text{//}\widehat{\text{x}}$, $\overrightarrow{\text{E}}\text{//}\widehat{\text{y}}$, $\overrightarrow{\text{E}}\text{//30}^{\text{o}}$, LCP and RCP wave.


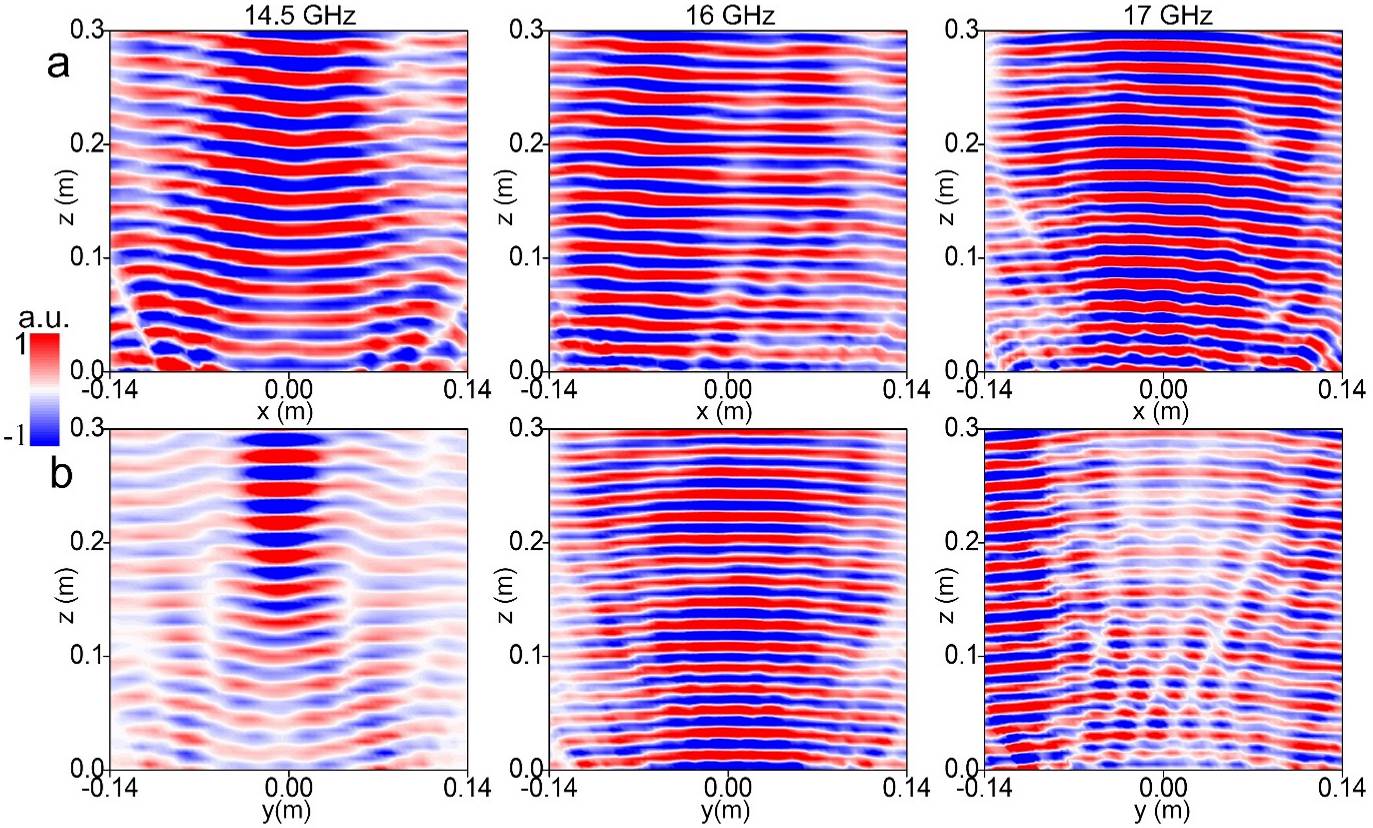


**Fig. S10** Experimentally measured NF *E*_x_ distributions in (a) xz and (b) yz cross-section plane of the pyramid conformal-skin cloak at different frequencies of 14.5, 16 and 17 GHz under normal incidence with $\overrightarrow{\text{E}}\text{//}\widehat{\text{x}}$.


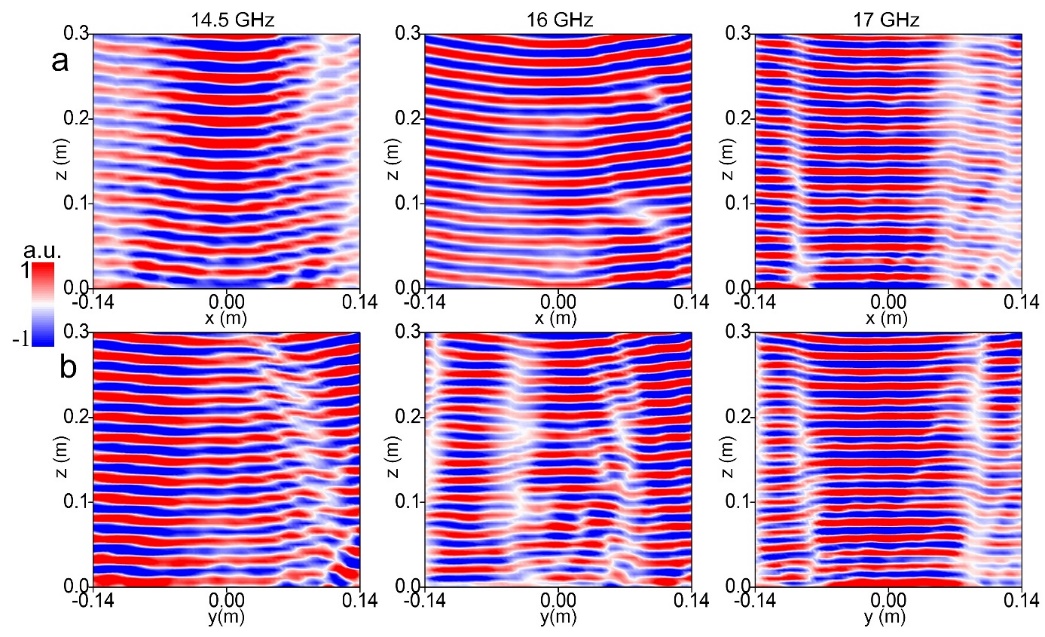


**Fig. S11** Experimentally measured NF *E*_y_ distributions in (a) xz and (b) yz cross-section plane of the pyramid conformal-skin cloak at different frequencies of 14.5, 16 and 17 GHz under normal incidence with $\overrightarrow{\text{E}}\text{//}\widehat{\text{y}}$.


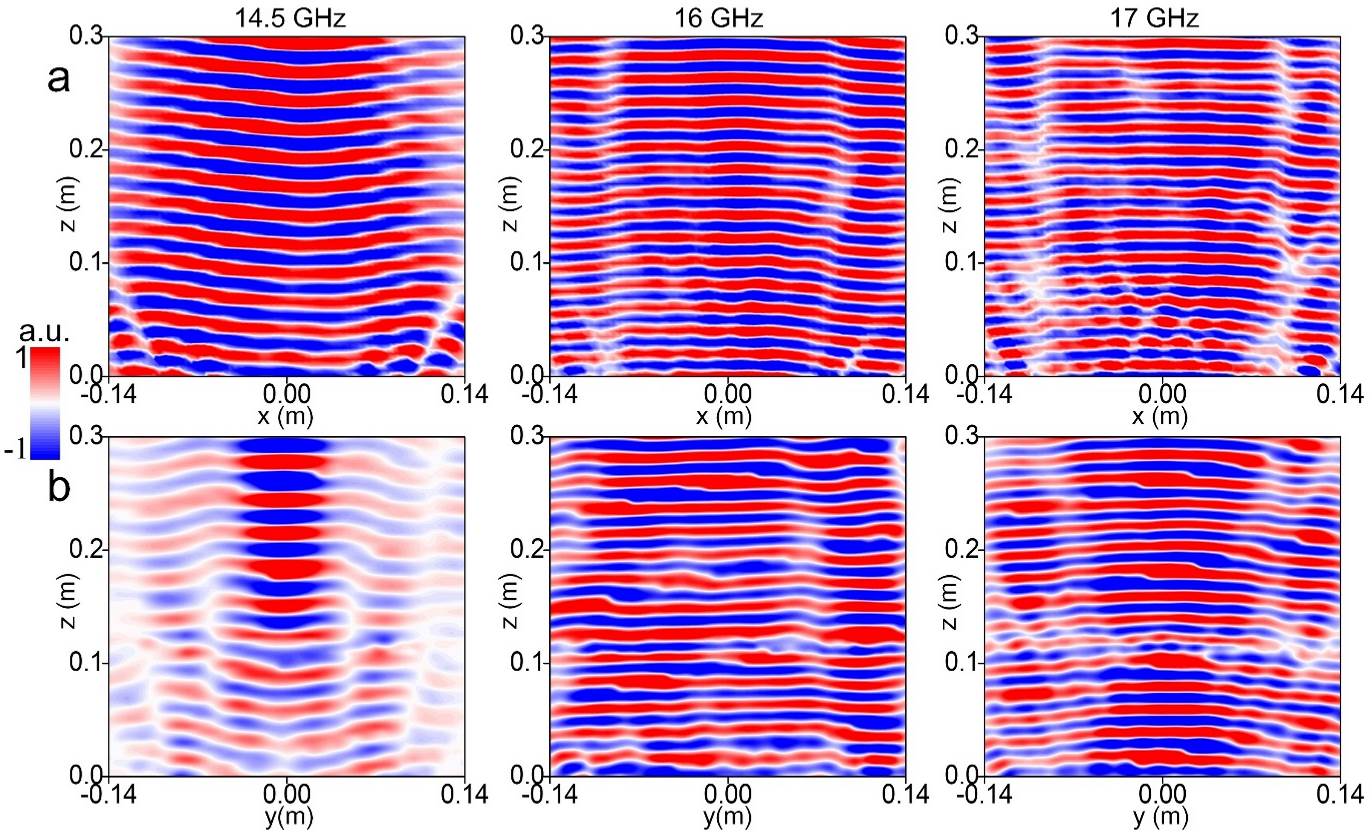


**Fig. S12** Experimentally measured NF *E*_x_ distributions in (a) xz and (b) yz cross-section plane of the pyramid conformal-skin cloak at different frequencies of 14.5, 16 and 17 GHz under LCP wave of normal incidence.


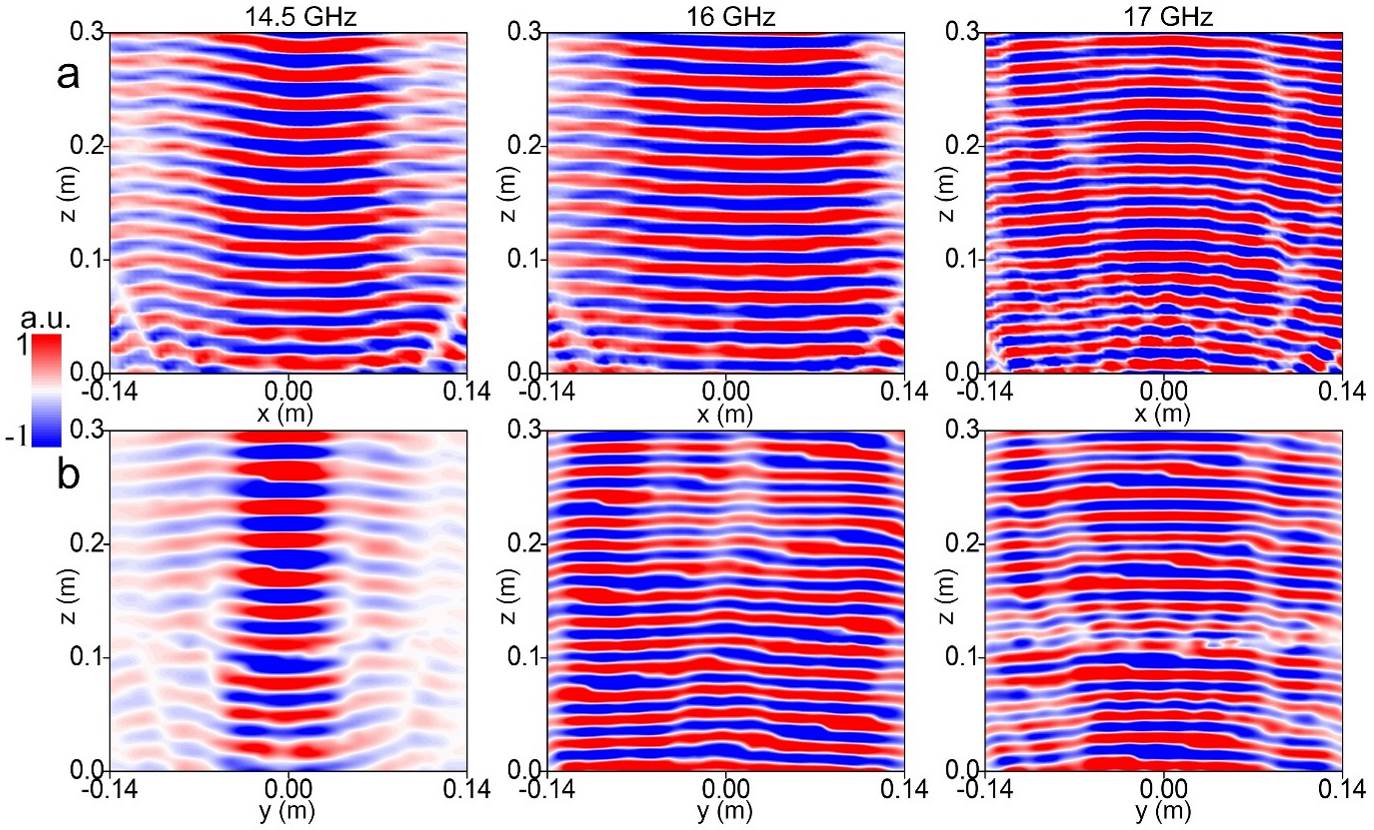


**Fig. S13** Experimentally measured NF *E*_x_ distributions in (a) xz and (b) yz cross-section plane of the pyramid conformal-skin cloak at different frequencies of 14.5, 16 and 17 GHz under RCP wave of normal incidence.


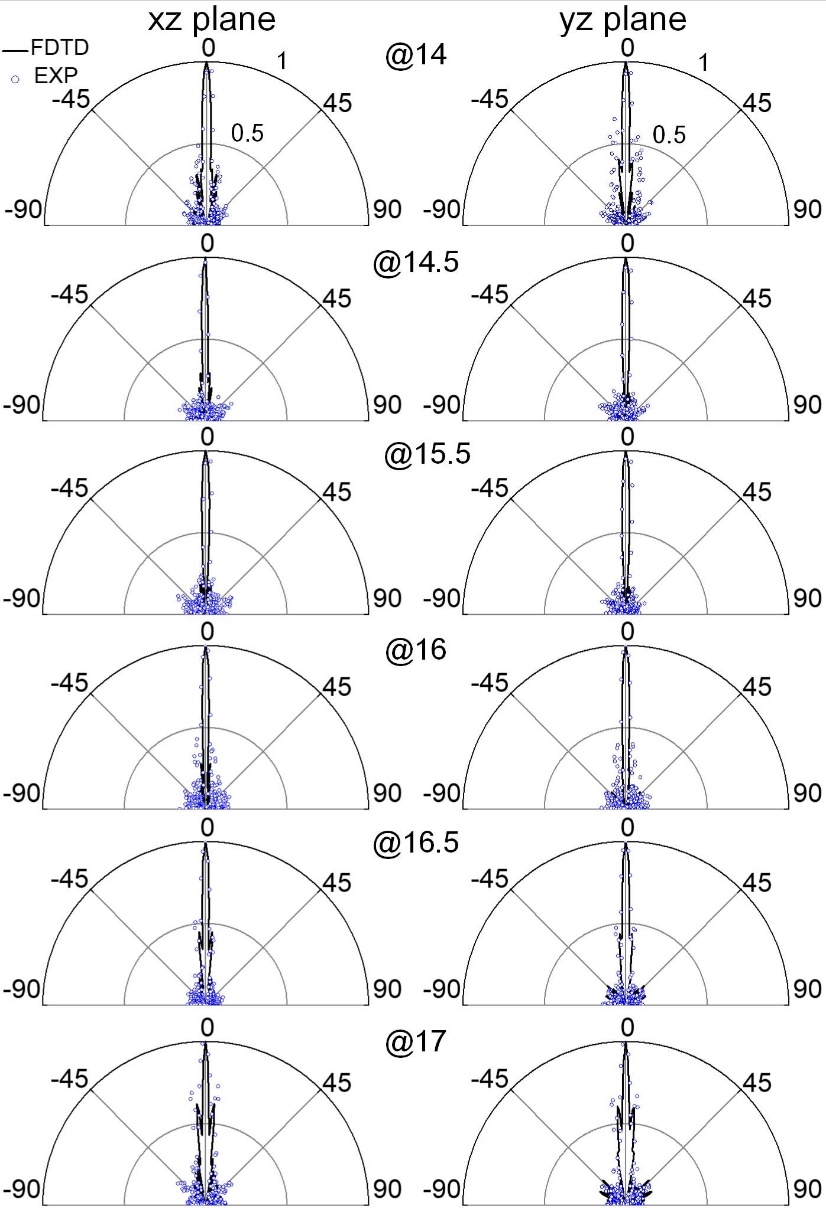


**Fig. S14** FDTD calculated and experimentally measured co-polarized FF scattering patterns in xz and yz cross-section planes of the pyramid conformal-skin cloak at different frequencies from 14 to 17 GHz in steps of 0.5 GHz under normal incidence with $\overrightarrow{\text{E}}\text{//}\widehat{\text{x}}$.

1. **Clocking performance of the pyramid conformal-skin cloak at oblique incidence**

In the main text, we evaluated the cloaking performance of the pyramid cloak at normal incidence. Here, we further check the angle-adaptive invisibility performance of the cloak at oblique incidence still in xz plane (º). As is much appreciated from Fig. S15, the pyramid cloak preservers the its mirror FF scattering patterns for *θ*_i_=-20^o^ and *θ*_i_=-30^o^ at four representative frequencies with negligible sidelobes. Moreover, the cloaking performances also deterioriate to some extent in terms of large sidelobes, wider beam and reduced bandwidth, this is especially true for the scattering pattern obtained at 14.5 GHz and *θ*_i_=-30^o^. Nevertheless, all results indicate an elegant cloaking behaviour with a bandwidth of 2.5 and 2 GHz for *θ*_i_=-20^o^ and *θ*_i_=-30^o^, respectively.


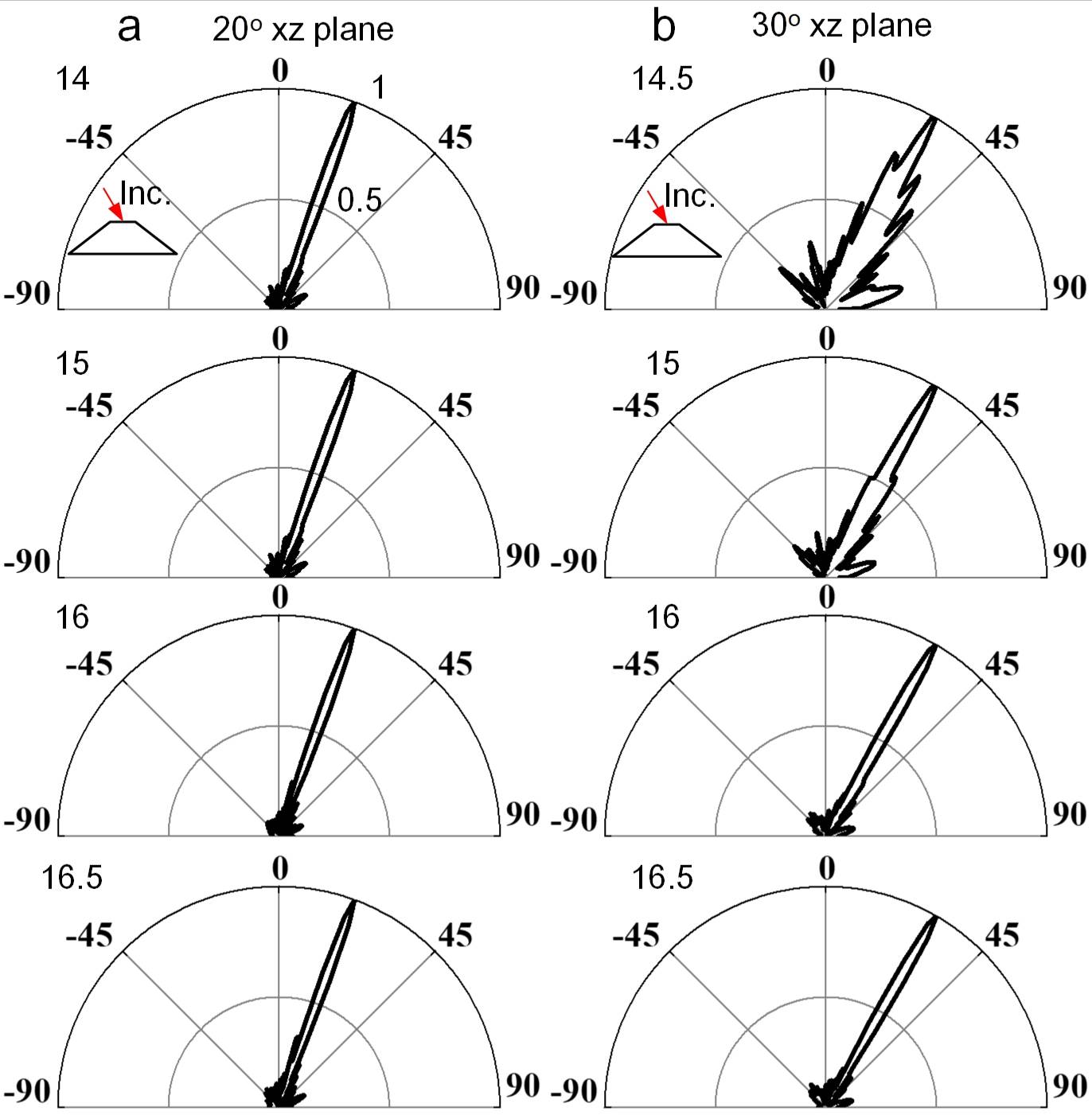


**Fig. S15** FF scattering patterns of the pyramid cloak at oblique incidence of *θ*_i_=-20^o^ and *θ*_i_=-30^o^ in xz plane at four representative frequencies. Similar scattering performance is achieved when EM wave is incident along -20^o^ and -30^o^ in yz plane.

As a step further, we examine its cloaking performance under illumination of a EM wave at arbitrary azimuthal angle () unde oblique incidence (*θ*) to illustrate the real 3D operation of our pyramid cloak. Two excitation cases with grouped azimuthal and elevation illumination angles are selected as an example, i.e., º and *θ*=10º, º and *θ*=20º. In these cases, the cloak are illuminated under non-symmetric illumination. Fig. S16a plots the illumination setup with arbitary and *θ* in FDTD calculation while Figs. S16b and S16c show their far-field scattering behavior. As expected, similar mirror reflection behavior with a pure main beam precisely directed toward predesigned angles is clearly observed in both cases in a desirable bandwidth, clearly indicating the real 3D operation for full azimuthal illumination angle.


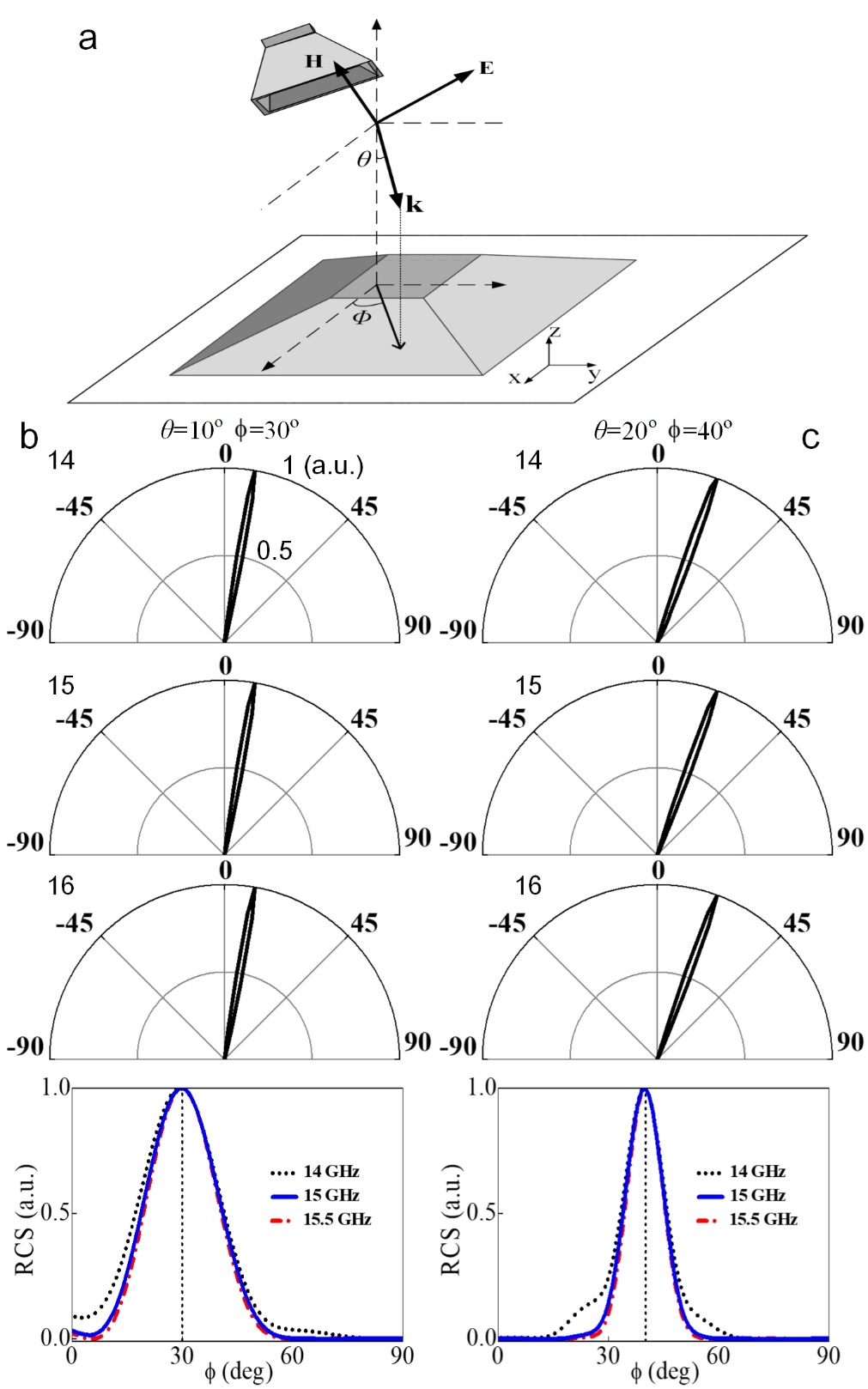


**Fig. S16** FF scattering patterns of the pyramid cloak at arbitrary azimuthal angle () unde oblique incidence (*θ*) at three representative frequencies of 14, 15 and 16 GHz. (a) Illumination setup. (b) FF scattering patterns at º plane and the maximum scattering intensity at *θ*=10º (bottom panel). (c) FF scattering patterns at º plane and the maximum scattering intensity at *θ*=20º (bottom panel).

1. **Characterization of other conformal-skin cloaks using different approaches**

To understand that synergizing the cross-LP dynamic and geometric phases serves as the only option to preserve the output polarization state of our proposed skin metasurface cloak, in both LP and CP modes, here we afford additional real cloak designs and FDTD calculations to strongly support this claim.

***A. Co-LP dynamic-phase and geometric-phase design***

In the first example, we designed a metasurface cloak by synergizing the co-LP dynamic and geometric phases based on similar meta-atoms employed in the main text. As a consequence, we utilize this equation to synthesize the required $\varphi_{xx}, \varphi_{yy}$ and *α*. The major difference lies in that here the Co-LP meta-atom is aligned along x or y axis, whereas the Cross-LP meta-atoms in the main text is aligned along 45º and 135º. Again, to construct the meta-atom library we calculate the Co-LP phase response ($\varphi_{xx} and \varphi_{yy}$) for a set of meta-atoms with different *β* and fulfilling the condition of $\varphi_{yy}-\varphi_{xx}=180º$ and $| r_{yy}|=|r_{xx}|=1$. Finally, the metasurface layout can be mapped in a similar manner according to the synthesized $\varphi_{xx}, \varphi_{yy}$ and *α* and the meta-atom library. Here, triangle bump is utilized with structure parameters detailed in the caption of Fig. S17. The layout of above cloak by chance corresponds to that of a cloak without involving geometric phase pattern but by directly imposing the theoretically calculated cloaking phase patterns $\delta$ shown in Fig. 3a to the Cross-LP dynamic-phase $\varphi_{xy}$. The cloak is targeted at 14 GHz and is normally trigged by $\left| \left. \sigma_{x} \right\rangle\right.$ and $\left| \left. \sigma_{y} \right\rangle\right.$ wave, respectively. Again, we wrap our cloak over the triangle metallic bump (Fig. S17a) and analyze its invisible properties. As is shown in Fig. S17a, the meta-atom is oriented either along 45^o^ or 135^o^ in xy plane, As a consequence, the resulting metasurface cloak is a cross-LP system that cannot preserve it polarization property under LP waves. As expected in Figs. S17b and S17c, the co-polarization fields are completely distorted with several beams scattered into different directions in free space under $\left| \left. \sigma_{x} \right\rangle\right.$ and $\left| \left. \sigma_{y} \right\rangle\right.$ wave excitation which is totally different from the uniform and narrow specular scattering (an equivalence of flat ground) shown in Fig. 3 in the main text, convincing us an impressive non-cloaking property. Note that although the cross-polarization fields are well reconstructed with the same phase and amplitude as if the light were incident on a flat reflective ground for both $\left| \left. \sigma_{x} \right\rangle\right.$ and $\left| \left. \sigma_{y} \right\rangle\right.$ wave, the cloaked bump is still detectable for a shared receiving/transmitting system.


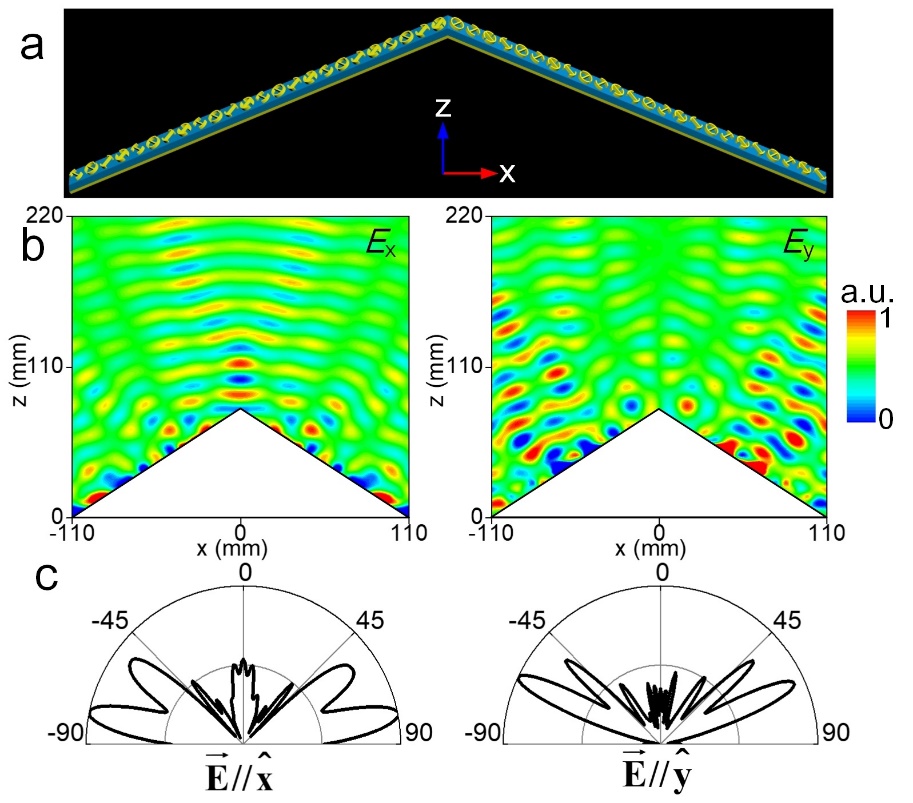


**Fig. S17** Illustration of the inefficiency of conformal-skin cloak based on Co-LP dynamic and geometric phase scheme or cross-LP dynamic-phase approach. (a) Layout of the triangle metasurface cloak composed of 1×48 meta-atoms. The triangle bump is with a tilt angle of *ψ*=30^o^ and a cross section of *L*×*H*=228.6 mm×68.5 mm. There are a total of 24 meta-atoms along each slope of the cloak. (b) FDTD calculated co-polarization NF and (c) FF results at 14 GHz when the cloaked bump is illuminated by a normally incident $\left| \left. \sigma_{x} \right\rangle\right.$ and (left panel) and $\left| \left. \sigma_{y} \right\rangle\right.$ (right panel) wave. Here, only the reflected field is plotted in NF patterns for clarity by subtracting the total field from the incident one. All NF and FF results are normalized to their maximum.

***B. Isotropic Co-LP dynamic-phase design***

To illustrate the inefficiency of available dynamic-phase approach based on isotropic co-LP meta-atom, here we further design a real metasurface cloak (Fig. S18a) composed of spatially varied double-ring meta-atom according to the phase distribution shown in Fig. S18b. The dynamic phase is realized by simultaneously changing the side length of the double-ring along x and y directions, see Fig. S18c. To not lose generality, the cloak wrapping over a bump with arbitrary conformal boundary is designed at the target frequency of 13 GHz. The bump is described by a piecewise function composed of three specific sections. The 3D printing polymer material ABS-M30 with a thickness of 3 mm, dielectric constant *ε*_r_=2.7 and loss tangent tanδ=0.005 is utilized as dielectric spacer. Since there is an obvious dual-fold rotational symmetry and mirror symmetry, the meta-atom is in isotropic co-LP scheme and the resulting cloak works under dual LP states, similar to [1]-[3]. Here we concentrates on the cloaking performance of the cloak on dual CP states. Therein, we adopt plane $\left| \left. \sigma_{+} \right\rangle\right.$ and $\left| \left. \sigma_{-} \right\rangle\right.$ wave to normally trigger the cloak. As expected in Figs. S18d and S18e, the *E*_y_ fields and co-polarization FF patterns are completely distorted with several beams scattered into different directions in free space under $\left| \left. \sigma_{+} \right\rangle\right.$ and $\left| \left. \sigma_{-} \right\rangle\right.$ wave excitation which is totally different from the uniform and narrow specular scattering of a flat ground, convincing us an impressive non-cloaking property. As a consequence, the cloak based on dynamic-phase approach cannot preserve its polarization under CP wave excitations by employing co-LP meta-atoms.

***
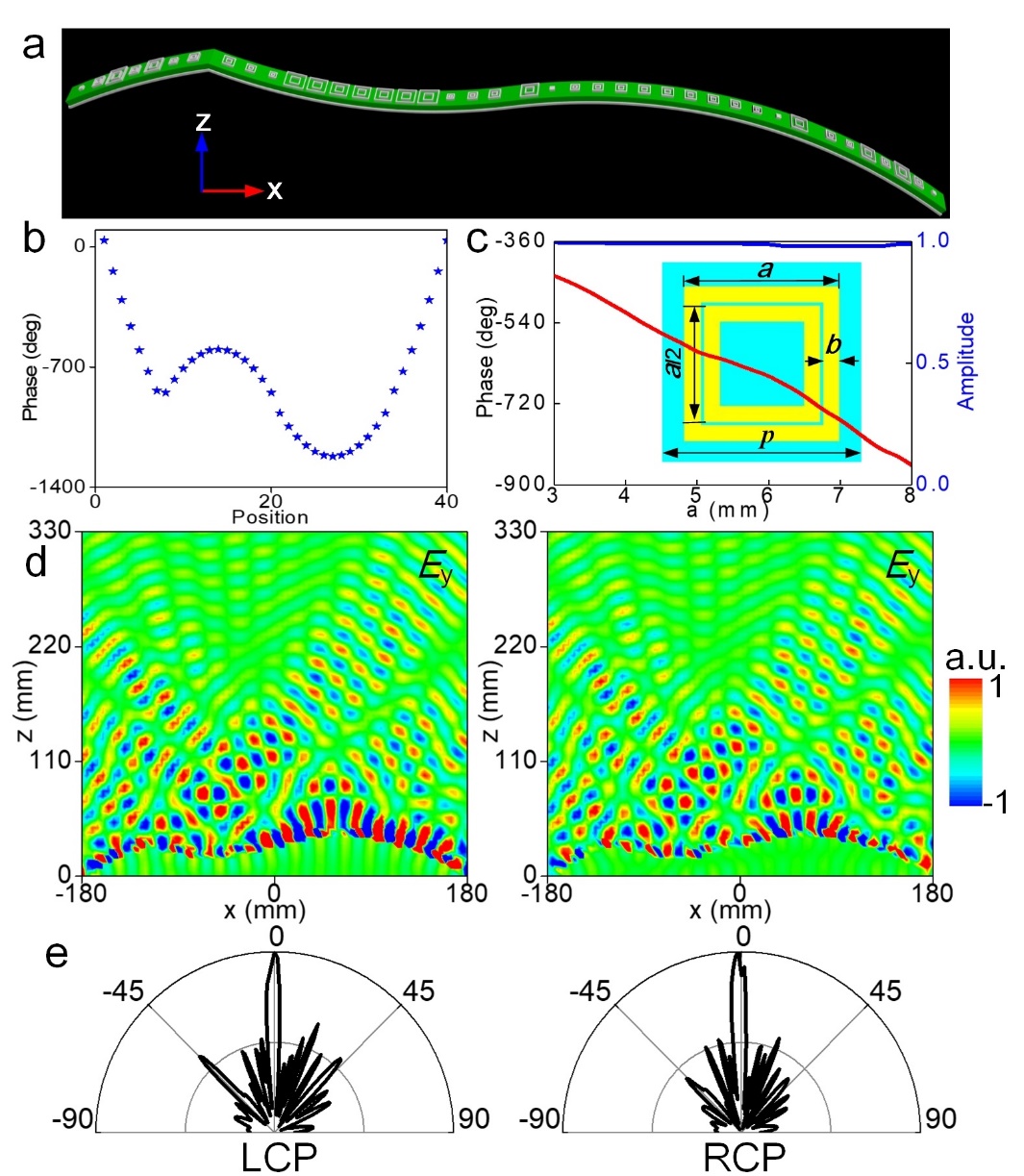
***

**Fig. S18** Illustration of the inefficiency of metasurface cloak in co-LP scheme based on available dynamic–phase approach. (a) Layout of the undulate metasurface cloak composed of 1×40 meta-atoms and occupied a cross section of *L*×*H*=360 mm ×9 mm. The undulate bump is described by a piecewise function composed of three specific sections:$z=-\frac{1}{360}(x+60)^{2}+40\text{ }(-180\leq x\leq-120)$,$z=60+\frac{1}{360}(x+60)^{2}-40\text{ (}-120\leq x\leq0\text{)}$, $z=-\frac{1}{360}(x-60)^{2}+40\text{ }(0\leq x\leq180)$. There are a total of 7, 13 and 20 meta-atoms along each section. (b) Theoretically calculated cloaking phase patterns. (c) Reflection amplitude and phase response of the meta-atom as a function of side length *a*, the inset illustrates the detailed parameters which are *b*=0.6 and *p*=9 mm. FDTD calculated (d) NF (*E*_y_) and (e) co-polarization FF results at 12.8 GHz when the cloaked bump is illuminated by a normally incident $\left| \left. \sigma_{+} \right\rangle\right.$ (left panel) and $\left| \left. \sigma_{-} \right\rangle\right.$ (right panel) wave. Here, only the reflected field is plotted in NF patterns for clarity by subtracting the total field from the incident one. All NF and FF results are normalized to their maximum.

1. **Sample fabrication and experimental setup**

Figs. S19 and S20 illustrate the sample fabrication process, and the NF and FF experimental setup. The detailed description of the sample fabrication and experimental setup can be seen from the Methods in the main text.

**
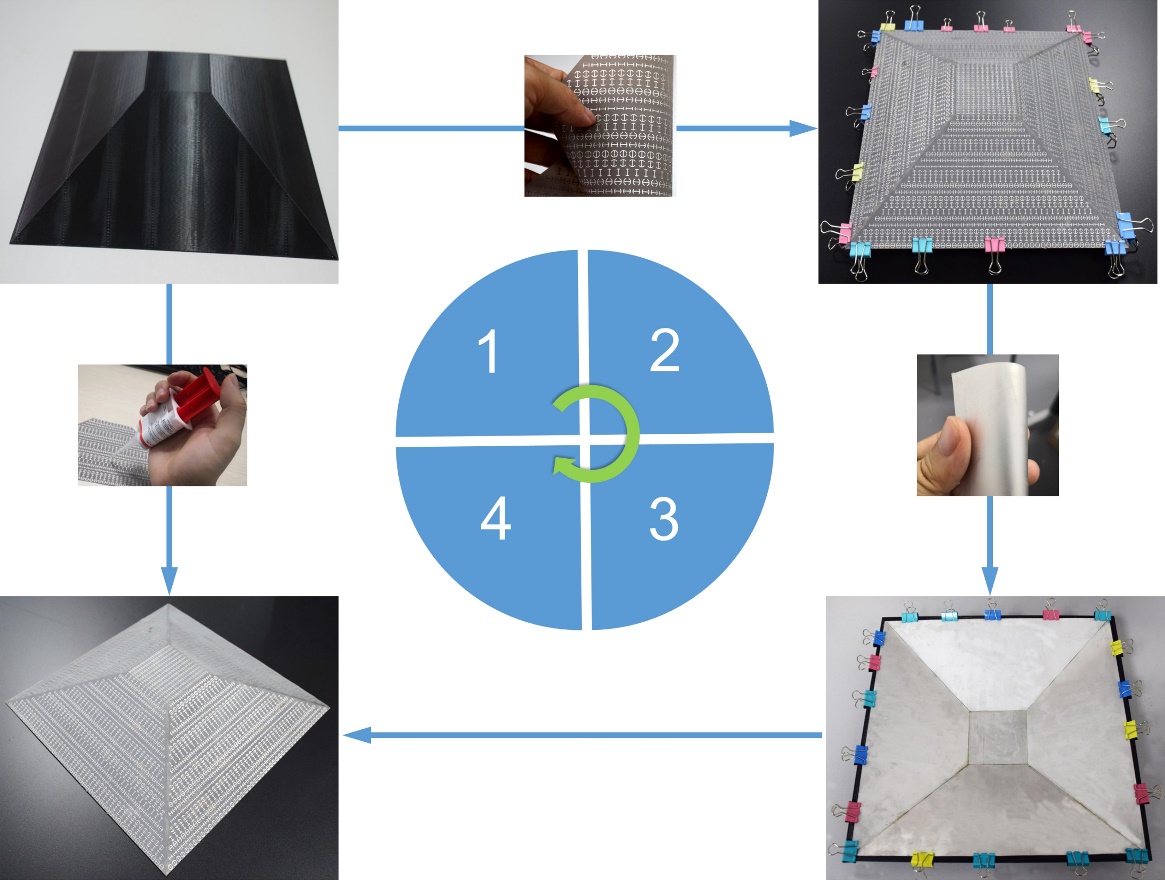
**

**Fig. S19** The fabrication process of our full-polarization conformal-skin cloak. The cloak sample is designed using a computer-aided design (CAD) process and prepared based on four-step dual-sided fabrication process by combining 3D-printing and flexible printed circuit board (PCB) technique.


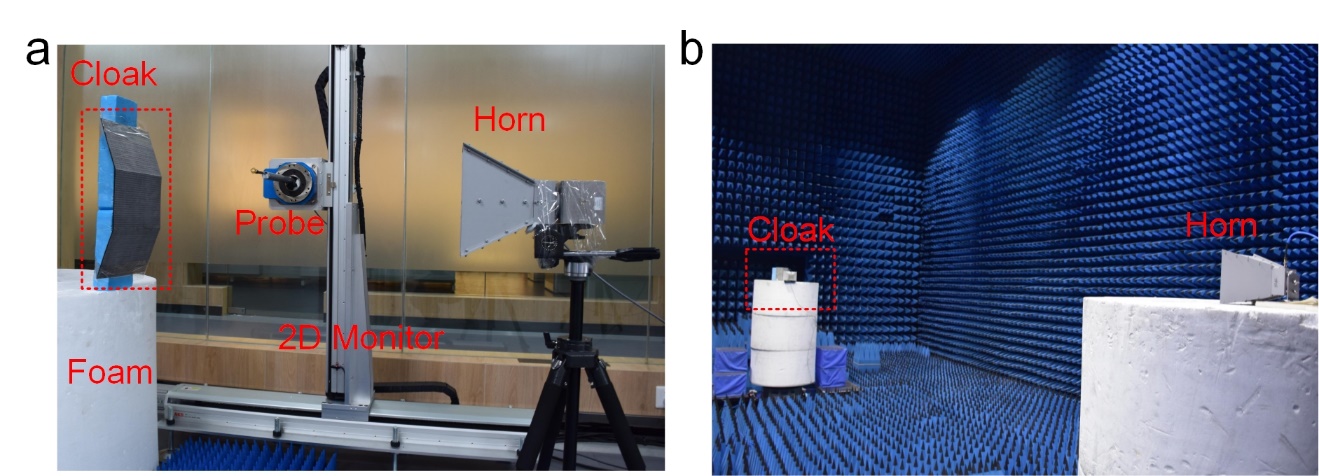


**Fig. S20** (a) NF and (b) FF experimental setup for the full-polarization conformal-skin cloak.

**References**

1. Yang, Y. et al. Full-polarization 3D metasurface cloak with preserved amplitude and phase. *Adv. Mater.* **28**, 6866–6871 (2016).
2. Orazbayev, B., Mohammadi Estakhri, N., Alu, A. & Beruete, M. Experimental demonstration of metasurface-based ultrathin carpet cloaks for millimeter waves. *Adv. Opt. Mater.* **5**, 1600606 (2017).
3. Jiang, Z. J. et al. Experimental demonstration of a 3D-printed arched metasurface carpet cloak. *Adv. Opt. Mater*. **7**, 1900475 (2019).
